# α-Synuclein interacts directly but reversibly with psychosine: implications for α-synucleinopathies

**DOI:** 10.1038/s41598-018-30808-9

**Published:** 2018-08-20

**Authors:** Hazem Abdelkarim, Michael S. Marshall, Giuseppe Scesa, Rachael A. Smith, Emily Rue, Jeffrey Marshall, Vince Elackattu, Monika Stoskute, Yazan Issa, Marta Santos, Duc Nguyen, Zane Hauck, Richard van Breemen, Maria S. Celej, Vadim Gaponenko, Ernesto R. Bongarzone

**Affiliations:** 1 Department of Biochemistry and Molecular Genetics, College of Medicine, University of Illinois at Chicago, Chicago, IL 60607 USA; 2 Department of Anatomy and Cell Biology, College of Medicine, University of Illinois at Chicago, Chicago, IL 60612 USA; 3 Department of Medicinal Chemistry and Pharmacognosy, College of Pharmacy, University of Illinois at Chicago, Chicago, IL 60612 USA; 4 Departamento de Química Biológica Ranwel Caputto, and Centro de Investigaciones en Química Biológica de Córdoba, CIQUIBIC, CONICET, Universidad Nacional de Córdoba, Córdoba, Argentina; 5 Departamento de Química Biológica, IQUIFIB, Universidad Nacional de Buenos Aires, Buenos Aires, Argentina

## Abstract

Aggregation of α-synuclein, the hallmark of α-synucleinopathies such as Parkinson’s disease, occurs in various glycosphingolipidoses. Although α-synuclein aggregation correlates with deficiencies in the lysosomal degradation of glycosphingolipids (GSL), the mechanism(s) involved in this aggregation remains unclear. We previously described the aggregation of α-synuclein in Krabbe’s disease (KD), a neurodegenerative glycosphingolipidosis caused by lysosomal deficiency of galactosyl-ceramidase (GALC) and the accumulation of the GSL psychosine. Here, we used a multi-pronged approach including genetic, biophysical and biochemical techniques to determine the pathogenic contribution, reversibility, and molecular mechanism of aggregation of α-synuclein in KD. While genetic knock-out of α-synuclein reduces, but does not completely prevent, neurological signs in a mouse model of KD, genetic correction of GALC deficiency completely prevents α-synuclein aggregation. We show that psychosine forms hydrophilic clusters and binds the C-terminus of α-synuclein through its amino group and sugar moiety, suggesting that psychosine promotes an open/aggregation-prone conformation of α-synuclein. Dopamine and carbidopa reverse the structural changes of psychosine by mediating a closed/aggregation-resistant conformation of α-synuclein. Our results underscore the therapeutic potential of lysosomal correction and small molecules to reduce neuronal burden in α-synucleinopathies, and provide a mechanistic understanding of α-synuclein aggregation in glycosphingolipidoses.

## Introduction

α-Synuclein is an amyloidogenic protein involved in synucleinopathies, such as Parkinson’s disease (PD), Lewy body dementia, and several lysosomal storage diseases (LSD), including Gaucher’s disease and Krabbe’s disease (KD)^1^. Despite their distinct etiology, the common feature of synucleinopathies is the formation of insoluble α-synuclein aggregates in brain neurons, suggesting that different mechanisms may converge to aggregate the protein^2,3^. α-Synuclein is a 140-amino acid (aa) protein encoded by the *SNCA* gene^4,5^, and encompasses three distinct regions: an amphipathic NH_2_-terminal region (aa_1–60_) that adopts an α-helical structure upon membrane binding; a non-amyloid-β component (NAC) region (aa_61–95_) involved in protein aggregation; and a highly acidic COOH-terminal region (aa_96–140_) that masks the NAC region and reduces α-synuclein aggregation^4,6^. Various mechanisms have been proposed for the aggregation process^7–12^, which involves exposure of the NAC region. α-Synuclein forms at least two types of aggregates: (i) non-toxic fibrillization-resistant tetramers^8,9^ observed under normal conditions; and (ii) toxic dimers, trimers, and higher-order oligomers assembling into insoluble fibrils^7,10^ in disease^13^.

The factor(s) influencing α-synuclein self-association into toxic or non-toxic forms is still unclear, but mounting evidence suggests a central role for lysosomal function and glycosphingolipids (GSL) in this process^14–19^. For example, deficient lysosomal activity correlates with α-synuclein aggregation in the PD brain^20,21^, and patients with mutations for Gaucher’s disease^22–29^, an LSD caused by a deficiency of glucosylceramidase activity, are at increased risk of PD. Genome-wide association studies recently identified the gene encoding the lysosomal enzyme galactosyl-ceramidase (GALC) as another risk factor for PD^30^. The first report of PD patients carrying allelic GALC mutations has been recently published^31^. Mutations in the GALC gene cause the toxic accumulation of psychosine in KD^32–37^. Psychosine, a GSL composed of a galactosyl sugar moiety bound to the sphingoid base sphingosine, is a potent inhibitor of several neuronal functions in KD^38–40^ and facilitates the formation of insoluble α-synuclein aggregates *in vitro*^1^. Aggregates of α-synuclein were found in neurons in the cortex, hippocampus, midbrain, striatum, and brainstem in the twitcher (TWI) mouse model of KD, as well as in the brain of affected human patients^1,41^.

The aggregation of α-synuclein in the context of lysosomal enzyme deficiencies presents the opportunity to measure the capacity of genetic corrections to prevent such aggregation in the brain, and to examine the molecular and structural mechanisms involved in the aggregation of α-synuclein by psychosine in particular and by GSL in general^42,43^.

Here, using multi-pronged genetic and NMR-based approaches, we show that the aggregation of α-synuclein can be prevented *in vitro* and in the brain of mutant mice deficient for GALC lysosomal activity. Furthermore, we find that psychosine facilitates α-synuclein aggregation via interaction between the sphingosine amino group with the negatively charged carboxy terminus of α-synuclein and the formation of galactosyl hydrophilic clusters. This interaction promotes the exposure of the NAC domain, leading to aggregation. Importantly, dopamine and carbidopa, currently used in PD treatment regimens, counter the conformational changes induced by psychosine and significantly reduce the formation of protein aggregates.

## Methods

### Materials and reagents

Stock solutions of psychosine (Matreya, Cat# 1305), N-acetyl-psychosine (Matreya, Cat# 1325), D-sphingosine, and N-hexanoyl-D-sphingosine (Matreya, Cat# 1809) were dissolved in 100% hexadeuterodimethyl sulfoxide (DMSO-d_6_, Sigma-Aldrich, Cat# 547239) per the manufacturers’ recommendations. Stock solutions of dopamine hydrochloride (Sigma-Aldrich, Cat# H8502) and carbidopa (Sigma-Aldrich, Cat# C1335) were dissolved in 100% Deuterium oxide (D_2_O, Sigma-Aldrich, Cat# 151882).

### Mouse models

Animal studies were performed in accordance with relevant guidelines and regulations contained in the approved protocols from the Animal Care and Use Committee of the University of Illinois at Chicago. Wild-type (*GALC*+/+) and twitcher mice (*GALC*−/−) were identified by PCR as described^44^. Twitcher mice (*GALC*−/+) were crossed with a strain of the same background (C57BL/J6), with the gene for α-synuclein (*SNCA*−/−) knocked-out. Littermate progeny with the genotypes TWI (*GALC*−/−*; SNCA*+/+), WT (*GALC*+/+*; SNCA*+/+), WT/SNCA−/− (*GALC*+/+*; SNCA*−/−), TWI/SNCA+/− (*GALC*−/−*; SNCA*+/−), and TWI/SNCA−/− (*GALC*−/−*; SNCA*−/−) were monitored for survival and a range of behavioral assays. The presence of the SNCA allele was detected as described^45^, with all mice maintained on a C57BL/6J background.

### Human brain samples

Fresh-frozen unidentified tissue was obtained from University of Maryland Brain and Tissue Bank. This work was performed under the guidelines and regulations for the use of de-identified human samples approved as an exempt study by the University of Illinois at Chicago Office for the Protection of Research Subjects. Fresh-frozen tissue to be used for biochemical analysis was microdissected using a sterile scalpel and kept frozen on dry ice during dissections.

### Disease severity score and survival

Progression of clinical signs was monitored using a scoring rubric developed in the lab, with a minimum score of 0 for no observable symptoms to a maximum score of 5 for the most severe signs. Mice were graded 3 times weekly by an observer blinded to the genotype. The observer measured weight loss (0 points, no weight loss; 0.5 points, 0–0.5 g weight loss; 1.0 point, 0.6–1.0 g weight loss; 1.5 points, 1.1–2 g weight loss; and 2.0 points, >2.0 g weight loss), presence of tremor (1.0 point for positive sign), and gait (0 points, normal gait; 1.0 points, waddling or any deviation from normal gait; 1.5 points, dragging limb; and 2.0 points, paralysis of limb or unable to ambulate). Mice that displayed a limb paralysis or 2 consecutive grading periods of >2.0 g weight loss were identified as near-terminal, and recorded at their maximal survival point.

### Grip Strength (Latency to fall)

Grip strength was assessed at P20, P30, and P40 (or until deceased). To test, mice were allowed to grasp edge of wire cage top with front paws. The observer (blinded to the animal’s genotype) then held the animal by the tail, parallel to the ground and recorded the time for the latency to let go from the cage. Maximum time measured was 45 seconds.

### αNesting Assay

On P20 before onset of motor symptoms, mice were individually housed overnight in an untorn cotton nestlet, which was scored the next morning for percentage (by weight) of nestlet shredded and architectural quality as follows: score of 1, <10% torn; score of 2, 10–50% torn; score of 3, shredded bedding not compiled into nest; score of 4, >50% torn, shredded bedding not compiled into a nest or flat nest but <50% total bedding shredded; score of 5, shredded bedding collected into a discernable nest, but flat without any walls built, and >50% shredded or shredded bedding collected into a discernable nest with walls built above 2 cm, but <50% total bedding shredded.

### NSC-34 cell culture, transfection and quantification of α-synuclein aggregates by bimolecular fluorescence complementation

Motorneuron cell line NSC-34 was used to study aggregation of α-synuclein using bimolecular fluorescence complementation (BiFC) to directly image α-synuclein oligomerization and aggregation. NSC-34 cells were seeded on poly-lysine-coated glass coverslips in 24-well plates (10,000 cells/cm^2^) and cultured in 10% FBS DMEM 0.5% penicillin/streptomycin. After 24 hours, cells were transfected with GN-linkaSYn and α-Syn-GC BiFC plasmids^46^ using Lipofectamine 2000 (ThermoFisher Scientific), according to manufacturer’s protocol. Briefly, for each sample, 1 μg of total plasmid DNA (50:50 molar ratio) and 3 μl of Lipofectamine were both diluted and mixed in 100 μl of DMEM, incubated for 5 min at room temperature, and added to cell cultures. Transfection reagents were removed with fresh proliferation medium 6 hours later. At 48 hours after transfection, culture medium was changed and the cells treated with 1 μM psychosine, 1 μM psychosine + 10 μM dopamine, 1 μM psychosine + 10 μM carbidopa, 10 μM dopamine, 10 μM carbidopa and 10 μM sphingosine for 2 days before fixation. Complementation was detected by fluorescence using confocal microscopy. BiFC+ aggregates per cell were counted and aggregate size was calculated using measuring tools in ImageJ software.

### Lysosome/autophagosome enrichment

Frozen whole-brain material from Twitcher and wild-type mice was processed using a lysosome enrichment kit for tissue and cultured cells (cat# 89839, ThermoFisher Scientific). Briefly, tissue was homogenized using a T8.10 disperser polytron tissue homogenizer (IKA Works, Inc., Wilmington, NC) before organelles were isolated according to the manufacturer’s instructions. Protease inhibitors were added to the solution before homogenization (cat# 88668, ThermoFisher Scientific). Centrifugation was carried out using an Eppendorf 5810 centrifuge (Eppendorf, Hauppauge, NY) and a Beckman Coulter Optima L-100 XP ultracentrifuge with a MLS-50 rotor (Beckman Coulter, Brea, CA). Western blot analysis confirmed enrichment of lysosomes (Supplementary Fig. S1I).

### Psychosine quantitation

#### Chromatography

Separations were carried out on methanol-acetic acid lipid extracts using a Shimadzu (Kyoto, Japan) Nexera UHPLC system equipped with a Waters Acquity UPLC BEH amide column (2.1 mm × 50 mm, 1.7 µm). Psychosine was eluted using a 30-sec isocratic flow of 85% acetonitrile and 15% 5 mM ammonium formate and 0.2% formic acid in water at a flow rate of 0.90 mL/min. The injection volume was 1 µL, and the column temperature was 45 °C. Data acquisition and integration were carried out using Shimadzu Lab Solutions software. *Tandem mass spectrometry:* The UHPLC system was interfaced to a Shimadzu LCMS-8050 triple-quadrupole mass spectrometer equipped with positive ion electrospray and operated at unit resolution. Nitrogen was used for nebulization at a flow rate of 3.0 L/min, drying gas at 10 L/min, and heating gas flow at 10 L/min. The ion source capillary and vaporizer temperatures were 300 °C. Psychosine was measured using collision-induced dissociation and selected reaction monitoring (SRM). Argon was used as the collision gas at a pressure of 230 kPa. The SRM transition for psychosine was *m/z* 462 to 282, and the transition for the surrogate standard D-lactosyl-ß1-1′-D-*erythro*-sphingosine was *m/z* 624 to 282. The SRM dwell time was 50 msec.

### Immunofluorescence and stereology

Mice were anesthetized and perfused with saline followed by 4% paraformaldehyde before tissue was removed and processed for cryosectioning. Cryosections (30 μm) were blocked free-floating with blocking buffer (0.3 M glycine, 1% BSA, 5% normal donkey serum, 5% normal goat serum, 0.30% Triton X-100, TBS) for 1 hour at room temperature, followed by 24–72 hours of incubation at 4 °C with primary antibodies in blocking solution. After washing with TBS, tissue was incubated with secondary antibodies at room temperature for 1 hour in blocking solution and washed again in TBS. Finally, sections were incubated in 0.005% thioflavin-S (cat# T1892, Sigma, St. Louis, MO) for 8 min, followed by two washes in 50% ethanol for 1 min and a TBS wash. Tissue was mounted with Prolong Gold antifade reagent (cat# P36931 (Life technologies, Eugene, OR) and visualized using confocal microscopy (Leica TCS SPE, Wetzlar, Germany). Primary antibodies used included: LAMP-1 (cat# sc-19992, Santa Cruz, Santa Cruz, CA, 1:300 dilution), LC3A (cat# 4599 (Cell Signaling, Danvers, MA, 1:300 dilution), and α-synuclein (cat# 610787 (BD Biosciences, San Jose, CA, 1:300 dilution). Secondary antibodies were Cy3 anti-rabbit (cat #711-166-152, Jackson ImmunoResearch, West Grove, PA, 1:500 dilution), Alexa Fluor 546 anti-mouse (cat# A11030, ThermoFisher Scientific, 1:500 dilution), and Dylight 549 anti-rat (cat# 112-506-068 (Jackson ImmunoResearch, 1:500 dilution), Alexa Fluor 647 anti-mouse (cat# A21236, ThermoFisher Scientific, 1:500 dilution). Immunostaining of isolated lysosomes was performed by loading 10 μg of isolated lysosomes/autophagosomes onto poly-lysine-coated coverslips, fixed in 4% PFA for 30 min, washed with TBS, and blocked and stained as described above. For stereological quantification, three areas (dentate gyrus, deep layers of cerebral cortex, and the lateral posterior nucleus of the thalamus) were selected. Every other 50-μm section of serial coronal brain slices for each genotype were stained for thio-S. Quantification was performed with design-based stereology system (Stereo-Investigator version 8, MBF Bioscience, Williston, VT, USA), after tracing under 5X objective and counting under 63X objective (Zeiss AX10 microscope, Carl Zeiss Ltd., Hertfordshire, England). The sampling parameters were set up according to the software guide to achieve the coefficient of error ranged between 0.09 and 0.12 using the Gundersen test, normally with a counting frame size 100 × 100 µm, optical dissector height 35 µm, and an average of 10 sampling sites per section.

### Immunoblotting

Protein concentration of isolated lysosomes was determined using a BCA assay (cat# 23225. ThermoFisher Scientific), with 12.5 µg of protein lysate from TWI and WT input and lysosome-enriched samples loaded into 10-well 4–12% Bis-Tris protein gels (1.5 mm, cat# NP0335BOX, ThermoFisher Scientific). After gel electrophoresis, samples were transferred onto a PVDF membrane (cat# 1620177, Bio-Rad, Hercules, CA). Membranes were blocked with 5% milk/1%BSA in TBS-T for 1 hour at room temperature. Primary antibodies LAMP-1 (cat# sc-199992, Santa Cruz, 1:500 dilution), LC3A (cat# 4599, Cell Signaling, 1:1000 dilution), and α-synuclein (cat# 610787 (BD Biosciences, 1:1000 dilution)] were diluted in 1% BSA/TBS-T and incubated at 4 °C overnight. Secondary antibodies used were: anti-mouse HRP (cat# 7076, Cell Signaling, 1:4000 dilution), anti-rabbit HRP (cat# 7074, Cell Signaling, 1:4000 dilution), and anti-rat HRP (cat# 712-036-150, Jackson ImmunoResearch, 1:4000 dilution) all diluted in 5% milk/TBS-T and incubated on blots for 1 hour at room temperature. Blots were visualized on autoradiography film. For *in vivo* α-syn:psy calculation, 0.48 μg, 0.24 μg, 0.12 μg, 0.06 μg, and 0.03 μg of monomeric α-synuclein were run as a standard curve to calculate experimental α-synuclein amounts in lysate and lysosome/autophagosome fractions. For isolated total cell lysates and lysosome/autophagosome fractions, an equal volume of sample was used from each animal equal to the amount of lysate used for psychosine quantification. Specificity of α-synuclein antibodies was controlled using brain lysates of α-synuclein knockout mice as described^1^ and by pre-incubation with 1 mg/mL of purified α-synuclein monomer solution overnight at 4C.

### Quantitative PCR

mRNA was isolated from snap frozen Twitcher, WT, and AAV9-GALC treated TWI (at P40 and aged time points) brains using an ISOLATE II RNA Mini kit cat#BIO-52072 (Bioline USA Inc, Taunton, MA). mRNA concentrations were measured using a Nanodrop spectrophotometer (ND-1000) and then converted to cDNA with a High Capacity cDNA Reverse Transcription Kit cat#4368814 (ThermoFisher Scientific, Waltham, MA, USA). Real-time qPCR analysis was then performed using iQ SYBR Green Supermix cat#1708880 in an iQ5 real time PCR detection system (Bio-Rad, Hercules, CA, USA). Previously validated primers were selected from the Harvard Primer Bank (https://pga.mgh.harvard.edu/primerbank/) and synthesized from Sigma-Aldrich, Inc, (Saint Louis, MO). Primer sequences used: GAPDH (Forward Primer 5′-AGGTCGGTGTGAACGGATTTG-3′ and Reverse Primer 5′-TGTAGACCATGTAGTTGAGGTCA-3′), SCNA (Forward Primer 5′-GCAAGGGTGAGGAGGGGTA-3′ and Reverse Primer 5′-CCTCTGAAGGCATTTCATAAGCC-3′). Results were reported as fold changes respect wild-type levels, using GAPDH as a housekeeping gene control. Samples were run in triplicates and each genotype with n = 4 samples.

### Immunoprecipitation

Human tissue (2 infantile KD patients, 1 late-onset KD patient, and an infantile control) was obtained from the University of Maryland Brain Tissue Bank. Immunoprecipitation was performed using Dynabeads protein G (cat# 10007D, ThermoFisher Scientific) and a Dynamag-2 magnet (cat# 12321D, ThermoFisher Scientific). Initially, 50 µl of protein G beads were washed and incubated overnight at 4 °C with 5 µg of anti-α-synuclein (cat# MA1-90342, ThermoFisher Scientific) or normal mouse IgG1 (cat# 5415, Cell Signaling). Beads were washed and then incubated overnight at 4 °C with tissue lysate. Lysate was prepared from about 250 mg tissue from each sample homogenized in 30 volumes of sterile PBS with protease inhibitors (cat# 88668, ThermoFisher Scientific) using a T8.10 disperser polytron tissue homogenizer (IKA Works, Inc., Wilmington, NC). After incubation, lysate was removed and beads were washed 3 times with 200 μl PBS and transferred to a clean tube. PBS buffer was then removed and beads were used for psychosine extraction or protein elution.

### Transmission electron microscopy

Tissue was fixed with 4% paraformaldehyde/2.5% glutaraldehyde and embedded in Araldite. Ultra-thin sections (60 nm) were processed for transmission electron microscopy. Tissue was transferred to carbon-coated 400-mesh nickel grids and negatively stained with 2% w/v uranyl acetate. Grids were viewed using a 120-kV JEOL JEM-1220 equipped with a Gatan Es1000W 11 MP CCD camera. Immunoelectron microscopy was performed with free-floating penetration of primary antibodies, followed by Aurion (0.8 nm) gold secondary antibodies.

### Expression, purification and labeling of α-synuclein

Recombinant full-length and truncated (1–108) α-synuclein with and without ^15^N enrichment were expressed in *E. coli* (BL21-DE3) and purified as described^47^. Five microliters of pT7-7 plasmids containing the human α-synuclein insert were transformed into 100 μl of BL21-DE3 cells. Positive transformants were grown overnight, inoculated into 1 L of LB or M9 media supplemented with ^15^NH_4_Cl, and grown until the optical density reached 0.6 at 600 nm. Expression of α-synuclein was induced with 0.5 mM (final concentration) of IPTG. After 4 hours, cells were harvested by centrifugation at 5000 rpm for 15 min. The pellet was resuspended in 10 mM Tris-HCl, pH 8, 1 mM EDTA, 1 mM PMSF and frozen with liquid nitrogen. Cells were lysed by thawing in a 70 °C water bath and sonication. After centrifugation to remove cell debris, DNA was precipitated by streptomycin sulfate (10 mg/mL), and α-synuclein, by ammonium sulfate (361 mg/mL). The protein pellet was resuspended in 25 mM Tris-HCl, pH 7.5, and α-synuclein was purified by ion-exchange chromatography on the Akta Purifier System (GE Healthcare) equipped with a POROS HQ column (Applied Biosystems) and Unicorn 5.11 software. Truncated (1–108) α-synuclein was recovered in the flow-through of anion-exchange chromatography, loaded onto a Superdex 200 10/300 (GE Healthcare Life Sciences) column equilibrated with 25 mM Tris-HCl, pH 7.5, and eluted at 0.5 mL/min. Proteins were quantified by measuring their absorbance using the coefficient of extinction at 275 nm of 5600 M^−1^ cm^−1^ (full-length protein) and 1490 M^−1^ cm^−1^ (truncated version). A protein aliquot was analyzed by SDS-PAGE on 4–12% Bis-Tris gel (Novex, Life Technologies cat#NP0335) and probed with monoclonal antibodies against human α-synuclein (BD Biosciences, cat#610786 and Cell Signaling, cat#2628) (Supplementary Fig. S1J). For improved resolution, the purified protein was electrophoresed on a 16% Tricine gel (Invitrogen cat# EC6695) and stained with Coomassie blue. Purity of the protein was assessed by SDS-PAGE and judged to be greater than 90%.

### One–dimensional proton NMR analysis of D-sphingosine

^1^H-NMR spectra of 10 and 600 µM of D-sphingosine in PBS (D-erythro-Sphingosine, Matreya, Cat# 1802) were recorded at 15 °C in 90% deuterium oxide (D_2_O, Sigma-Adlrich, Cat# 151882) and 10% DMSO-d_6_ using trimethylsilylpropanoic acid (TSP-d_4_, Sigma-Aldrich, Cat# 269913) as an internal standard. ^1^H NMR spectra of 5, 10, 200, 600, and 2400 µM of psychosine were recorded at 15 °C in 90% D_2_O and 5% DMSO-d_6_ using TSP-d_4_ as an internal standard. Data were processed and analyzed with the Topspin 3.8 or 4.0.1 NMR software.

### One–dimensional proton NMR analysis of Dopamine

1D^1^H-NMR spectra of 600 µM dopamine (Dopamine hydrochloride, Sigma-Aldrich, Cat# H8502) in PBS were recorded at 15 °C in 10% deuterium oxide (D_2_O, Sigma-Adlrich, Cat# 151882) using trimethylsilylpropanoic acid (TSP-d_4_, Sigma-Aldrich, Cat# 269913) as an internal standard. 1D ^1^H NMR spectra were recorded at 0.5, 24, 48, 72, 96, and 105 hours. The sample was stored at 4 °C between each time point to match the same conditions of dopamine and psychosine in presence of α-synuclein for 15 and 96 hours. As we did not notice any dopamine oxidation products, samples were kept open to air at r.t and monitored for any color change as a sign of oxidation. On day 16, an intense change in color and a precipitate were observed. The precipitate was separated and reconstituted in 100% DMSO-d_6_ (Sigma-Aldrich, Cat# 151874) and 1D ^1^H NMR spectra were recorded at 15 °C. Data were processed and analyzed with the Topspin 3.8 or 4.0.1 NMR software.

### Water ligand observed via gradient spectroscopy (WaterLOGSY) spectroscopy

All NMR spectra are referenced relative to TSP-d_4_, the arbitrary internal standard. The one–dimensional ^1^H WaterLOGSY NMR spectra of 20 µM recombinant α-synuclein with 600 µM psychosine in PBS containing 10% D_2_O and 600 µM TSP-d_4_ were recorded at 15 °C. Individual spectra of psychosine and α-synuclein were recorded under the same buffer conditions as negative controls. All experiments were performed on a Bruker 800-MHz NMR spectrometer. Data were processed and analyzed with Topspin 3.8 or 4.0.1 NMR software. Relative intensity ratios were calculated as signal intensity in presence or absence of α-synuclein/signal intensity in absence of α-synuclein.

### Heteronuclear single-quantum correlation (HSQC) spectroscopy

All two-dimensional ^1^H-^15^N HSQC experiments were carried out on a Bruker 900-MHz NMR spectrometer equipped with a cryogenic probe, and data were processed with NMRPipe and analyzed with NMRDraw^48^. Titration was carried out with 20 µM of α-synuclein in PBS. Measurements were performed at 15 °C at a pH 7.6 or 4.7. The ^1^H-^15^N α-synuclein assignments were based on published results^49^. Psychosine concentrations in the titration experiments were 10, 20, 40, 100, 200, 400, 600, and 1000 µM. Other ligands were tested at 600 µM. The mean chemical shift difference (Δδ_*NH*_) was determined using the following equation:

$${{\rm{\Delta }}{\rm{\delta }}}_{NH}=\sqrt{\frac{{({\rm{\Delta }}{\rm{\delta }}H)}^{2}+{({\rm{\Delta }}{\rm{\delta }}N)}^{2}/25}{2}}$$

where ΔδH and ΔδN are the differences between HN and ^15^N chemical shifts, respectively, of the bound and free protein and 25 is an arbitrary scaling factor for ^15^N nuclei. Chemical shift perturbations (CSPs) greater than the average plus 1 SD deviation (black line) and maximum CSP due to variability in spectra of α-synuclein were considered significant (orange line). Intensities were processed and analyzed using NMRPipe/NMRDraw software^48^.

The percentage change in intensity per residue was calculated at each ratio as an absolute value using the following equation:

$${\rm{Change}}\,{\rm{in}}\,{\rm{intensities}}\,( \% )=(({\rm{I}}^\circ -{{\rm{I}}}^{{\rm{L}}})/{\rm{I}}^\circ )\times 100$$

where I° is residue intensity in the synuclein-only control, and I^L^ is residue intensity in synuclein in the presence of psychosine or polyamine. Intensities greater than the average plus 1 SD (black line) (calculated in presence of psychosine) and orange lines (calculated between synuclein-only controls) were considered significant (orange line). For dissociation constant (K_d_) determination, differences in mean intensity were calculated by subtracting the mean intensity of signals at the C-terminus of α-synuclein control from the mean intensity of signals at the C-terminus of α-synuclein in the presence of psychosine gradient concentrations (10, 20, 40, 100, 200, 400, 600, 1000 µM). Data were analyzed using GraphPad Prism 5 with a non-linear regression saturation single binding site equation. Mean values and standard errors were fitted using a regular fit. For Hill coefficient calculations, ^15^N chemical shifts of residues Q109 and A140 were plotted against psychosine concentrations and analyzed using GraphPad Prism5. The final concentration of DMSO-d_6_ in samples containing psychosine and polyamines was less than 1%. For samples containing N-acetyl-psychosine, D-sphingosine, and N-Hexanoyl-D-sphingosine, DMSO-d_6_ final concentration was 10%v/v. α-Synuclein-only samples were run under identical buffer conditions of their respective ligands.

### Statistical analysis

Statistics and graphs were prepared with Prism 8 software (Graphpad Software Inc., La Jolla, Ca). Data were analyzed using a one-way ANOVA with a post hoc Tukey multiple comparison test, with Gaussian distribution and p-values < 0.05 considered significant. Graphs represent the mean of independent measurements with errors bars representing standard error of the mean. Variation was comparable between groups. Biochemical studies using animal tissue were performed with a minimum of n = 3 animals of each genotype. All behavioral studies were performed with an observer blinded to the genotype. All immunofluorescent imaging was performed on at least n = 3 subjects with technical replicates to confirm observations.

## Results

### Genetic knock-out of α-synuclein ameliorates the neurological phenotype of a mouse model for lysosomal GALC deficiency

**Figure 1:**
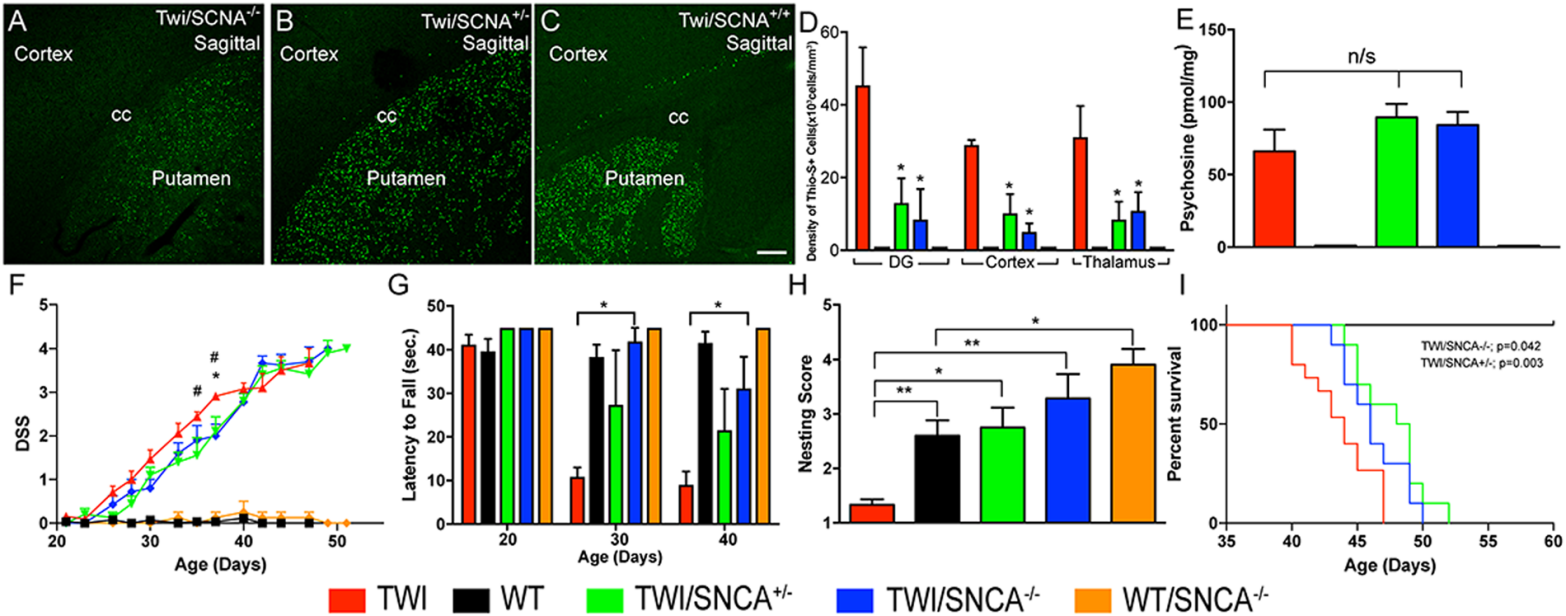
Knock-out of SNCA removes thioflavin-S-positive inclusions in certain brain regions and improves survival and behavior of TWI mice. (**A**) Brain tissue from TWI/SCNA−/− (**A**), TWI/SCNA+/− (**B**) and TWI/SCNA+/+ (**C**) were stained for thioflavin-S-positive protein aggregates. Thio-S material was visibly reduced in the lower cortical layers and putamen of TWI/SNCA−/− brains (**A**) and TWI/SNCA+/− brains (**B**) with respect to TWI/SNCA+/+ (**C**). Images (**A**–**C**) show anatomical regions encompassing deep cerebral cortex and the putamen. cc, corpus callosum. Scale bar = 200 μm. (**D**) Unbiased stereology quantitatively revealed a significantly reduced abundance of thio-S+ material in TWI/SNCA−/− and TWI/SNCA+/− brains (p = 0.001, ANOVA) within the Dentate Gyrus (DG), Cerebral Cortex (Cortex), and Thalamus. (**E**) Psychosine levels measured using LC-MS-MS showed non-significant (n/s) changes between the presence or absence of the SCNA allele in TWI brains. (**F**) A disease severity score (DSS) measured less severe signs in TWI/SNCA+/− and TWI/SNCA−/− mice compared to TWI/SNCA+/+, while changes were significant at only P36 and P38 for TWI/SNCA+/− (*p < 0.05) and P36 for TWI/SNCA−/− (*p < 0.05). (**G**) Grip strength, as assessed by a latency-to-fall test, was significantly improved in TWI/SNCA−/− compared to TWI/SNCA+/+ at P30 (*p < 0.05). (**H**) Nesting ability score, assessed at P20 in each genotype group, was significantly improved in both TWI/SNCA+/− and TWI/SNCA−/− compared to TWI mice (*p < 0.05, **p < 0.01, ANOVA). (**I**) Kaplan-Meier survival curves reveal significantly longer life of TWI/SNCA+/− and TWI/SNCA−/− compared to TWI. N = 10 mice (TWI/SNA^+/−^ and TWI/SNCA^−/−^) N = 15 mice (TWI/SNCA^+/+^).

We recently showed that the deficiency of lysosomal GALC activity in KD facilitates the formation of neuronal α-synuclein aggregates^1^, and that mutations in the GALC gene are associated with PD^41^. The first experimental question was to determine the contribution of α-synucleinopathy to the neurological phenotype in KD by ablating the expression of α-synuclein in the GALC-deficient TWI mouse. For this, α-synuclein SNCA^−/−^ null mice^50^ were used to produce TWI GALC^−/+^ SNCA^−/−^ carriers, which were viable and without any neurological phenotype. Progeny of GALC^−/−^ SNCA^−/−^ (TWI/SNCA^−/−^) pups were viable at birth and able to pass weaning. Removal of one or both alleles for α-synuclein visibly reduced the accumulation of thioflavin-S positive (thio-S+) material in several areas of the TWI/SNCA^−/−^ and TWI/SNCA^+/−^ brain, including cortex layers in the cerebrum, the putamen, and the hippocampus (Fig. 1A,B) in comparison to levels of thio-S+ material in TWI/SNCA^+/+^ mice (Fig. 1C). Stereological quantitation revealed significant reduction of thio-S+ material in these areas (Fig. 1D), consistent with our prior observations that aggregates of α-synuclein are part of a more complex folding defect affecting neurons in KD^1,41,42^. The knock-out of α-synuclein did not affect the metabolic blockage caused by the deficiency of GALC in TWI/SNCA^−/−^ mice, which accumulated psychosine at levels similar to those in TWI/SNCA^−/+^ and TWI/SNCA^+/+^ mice (Fig. 1E). However, knock-out of α-synuclein had a significant and positive impact on various clinical readouts, including disease severity score measured at several time points (Fig. 1F), latency to fall (Fig. 1G) and nesting skills (Fig. 1H) of mutant mice, suggesting that abnormal α-synuclein metabolism contributes to disease severity in GALC deficiency. While SNCA knock-out *per se* was insufficient to prevent early death, median animal survival was significantly extended beyond the mean of 42 days (Fig. 1I).

### Genetic correction of GALC deficiency normalizes psychosine metabolism and prevents the formation of α-synuclein and thio-S+ aggregates

**Figure 2:**
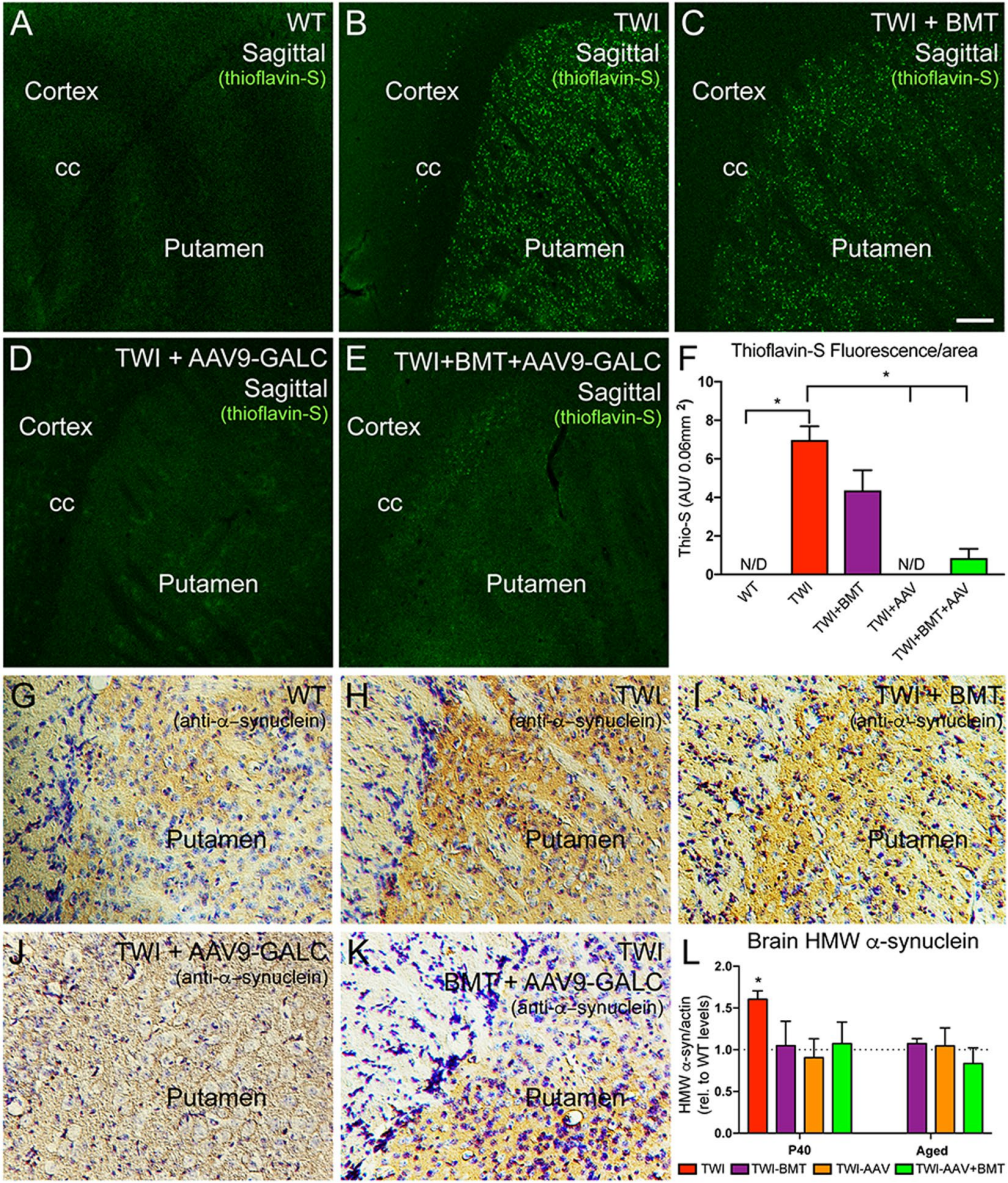
Neuronal thioflavin-S aggregates and high-molecular-weight α-synuclein aggregates are greatly reduced upon AAV9-GALC gene therapy. Thio-S+ protein aggregates in TWI (**B**), previously shown to be intra-neuronal and partly comprised of α-synuclein^1^, were eliminated at P40 after neonatal gene therapy using AAV9-GALC vectors alone^31^ (**D**) or in combination with bone marrow transplantation (BMT + AAV9-GALC) (**E**), but not by BMT alone (**C**). WT comparison in (**A**). Images show anatomical regions encompassing deep cerebral cortex and the putamen. cc, corpus callosum. Thio-S+ aggregates (fluorescent units per area) remained largely eliminated in AAV9-GALC and BMT + AAV9-GALC brains (**F**). *p < 0.05, compared to WT, ANOVA; n = 3–4 mice per group). (**G**,**K**) Gene therapy with AAV9-GALC greatly normalized α-synuclein levels in the cortex/putamen region (compare **J** and **K** with **H**). Sections were stained with diamino-bencidine for α-synuclein and counterstained with toluidine blue. (**L**) Total brain lysates from young (P40) and long-surviving (>P200) TWI, AAV9-GALC treated TWI and control mice^31^ were immunoblotted for α-synuclein, and high-molecular-weight bands (~30 to ~100 kDa) were quantified by optical densitometry. Results are expressed as fold levels over basal levels in WT brains. *p < 0.05, ANOVA; n = 3–4 mice per group. Scale bar: 200 μm.

In a recent study, we showed that neonatal gene therapy using adeno-associated viral vectors (AAV9) successfully corrected the lysosomal deficiency of GALC activity in TWI mice, leading to normalization of psychosine levels throughout the nervous system, prevention of neurological deficits, and almost complete normalization of life expectancy (~1200% increase over untreated mutants)^31^. That study provided the groundwork to investigate whether genetic correction of the lysosomal defect has an impact on the formation of thio-S+ material and α-synuclein aggregates in the brain of TWI mice. Brain material obtained in our prior study (Marshall *et al*. 2018a) was first examined for thio-S+ staining. Confocal analysis revealed the complete absence of thio-S+ deposits throughout the brain of TWI mice subjected to AAV9-GALC gene therapy. For example, the putamen of AAV9-GALC-treated TWI was completely devoid of thio-S+ aggregates (Fig. 2D,F) as compared to their presence in untreated TWI (Fig. 2B,F) and total absence in WT (Fig. 2A). By contrast with the results of AAV9-GALC therapy, treatment of TWI with bone marrow transplantation (BMT), the standard of care for KD^51,52^, minimally affected thio-S+ aggregates (Fig. 2C,F). The combination of BMT and AAV-GALC gene therapy was slightly less effective than gene therapy alone (Fig. 2E,F). The aggregation of α-synuclein detected in untreated TWI (Fig. 2H) appeared to be reduced after single-gene therapy for GALC in the TWI brain (Fig. 2J), whereas single BMT (Fig. 2I) or a combination of BMT and gene therapy was less efficient (Fig. 2K). WT levels of α-synuclein staining are shown for comparison (Fig. 2G). Reductions in α-synuclein aggregation were confirmed with western-blotting of higher-molecular-weight aggregates of α-synuclein in protein extracts showing a nearly complete normalization in the brain of young (postnatal day 40, P40) or aged single-gene therapy-treated TWI mice (quantified in Fig. 2L, original blots in Supplementary Fig. 18A–D and uncropped quality control for anti- α-synuclein blots in Supplementary Fig. 1G), indicating that normalization of the lysosomal metabolism of GALC and low levels of psychosine accumulated in the brain of treated TWI mice^31^ have a direct and positive preventive therapeutic effect on α-synuclein metabolism. qPCR analyses showing lack of increased α-synuclein mRNA production in the twitcher supports the notion that aggregation is due to fibrillization of existing protein rather than increased synthesis (Supplementary Fig. 1H).

### Psychosine and α-synuclein co-localize in the autophagosomal/lysosomal pathway in the GALC-deficient brain

**Figure 3:**
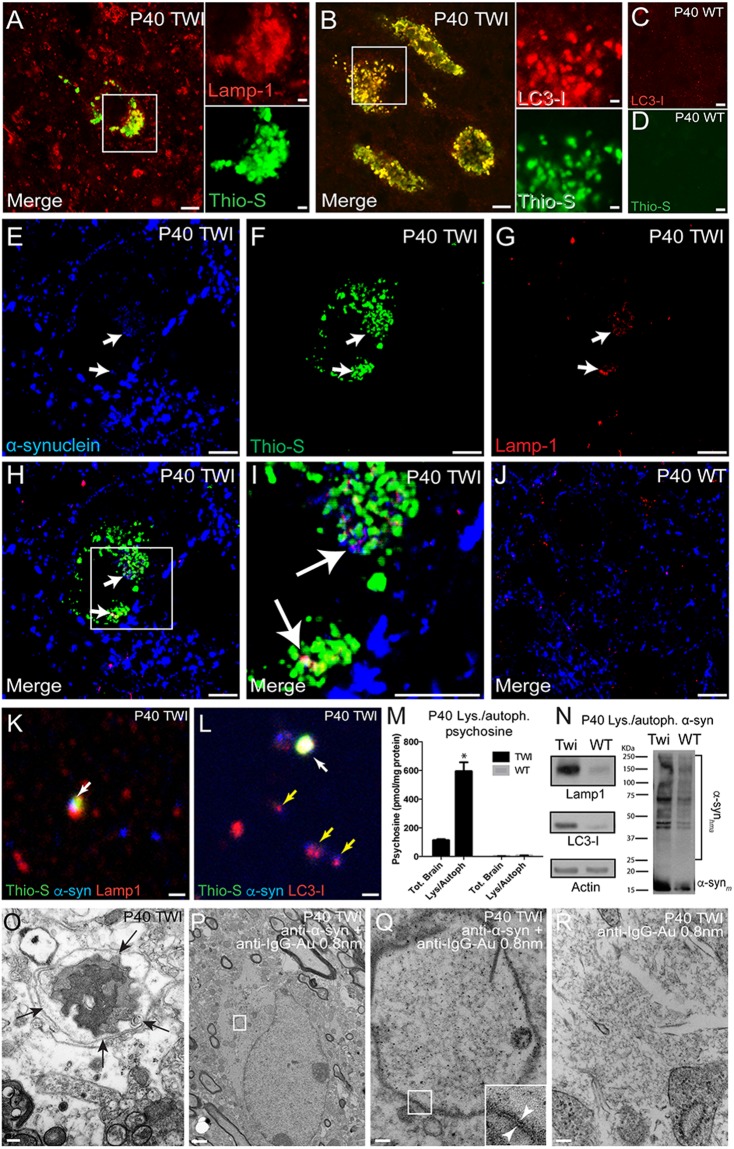
Psychosine and α-synuclein subcellular localization *in vivo*. Thioflavin-S (thio-S)-positive deposits in the P40 TWI brain co-localized with both the lysosomal marker Lamp-1 (**A**) and the autophagosomal marker LC3-I (**B**). TWI autophagosomes were enlarged compared to WT (**C**) and no aggregated thio-S material was detected in the WT brain (**D**). Anti-α-synuclein antibodies co-localized intracellularly with thio-S and the lysosomal marker LAMP-1 (**E**–**I**, arrows). Thio-S-positive α-synuclein was not detected in WT tissue (**J**). Isolation of lysosomes further confirmed α-synuclein co-localization with enriched lysosomes (**K**) and autophagosomes (**L**) while also staining positive for thio-S (**K**,**L** white arrows). Not all organelles that co-localized with α-synuclein were positive for thio-S (**L**, yellow arrows). Psychosine levels measured in total lysate and isolated lysosomes showed an accumulation in TWI that was significantly enriched in the lysosome/autophagosome fraction compared to the total lysate (n = 3, p < 0.05, ANOVA) (**M**). Fractions enriched in lysosomes and autophagosomes from TWI and WT brains showed an elevated level of Lamp-1 and LC3-I in addition to high-molecular-weight aggregates of α-synuclein in TWI (**N**). Ultrastructure EM imaging revealed fibrous material enveloped in apparent autophagic vacuoles (**O**, double membrane organelle marked by clack arrows), and immunoelectron micrography identified α-synuclein deposits within double-membrane (arrowheads, inset in **Q**) autophagosome structures (**P**,**Q**) in neurons from P40 TWI. Non-specific binding of secondary antibody (anti-IgG-Au, 0.8 nm) was not observed (**R**). Scale bars: 10 μm (**A**–**L**), 0.2 μm (**O**), 1 μm (**P**), and 100 nm (**R**). Uncropped blots displayed in Supplementary Fig. 18.

The results above and those from our previous study^1^ indicate that α-synuclein is sensitive to psychosine levels, consistent with a growing body of evidence showing the importance of GSL in the formation of α-synuclein aggregates. To investigate the mechanism involved in psychosine-mediated aggregation of α-synuclein, we first tested whether both molecules segregate in similar or different cell compartments. Neuronal thio-S+ deposits in TWI do not form large and solid structures classically seen in Lewy bodies, but rather display a punctate pattern throughout the cytoplasm of affected neurons (Fig. 3A,B). In fact, α-synuclein aggregates in TWI are not amenable to the traditional means of Lewy body isolation^53^ (data not shown). Confocal analysis showed co-localization of thio-S+ deposits with enlarged lysosomes and autophagosomes, as identified by anti-Lamp-1 and anti-LC3-I antibodies, respectively (Fig. 3A,B). Enlarged autophagosomes and thio-S+ material were absent in age-matched WT brains (Fig. 3C,D). Background controls with thio-S and secondary antibodies in WT tissue showed no accidental co-localization (Supplementary Fig. 1A,B). Because thio-S is not specific for misfolded α-synuclein, immunofluorescence analysis was performed using α-synuclein antibodies (Fig. 3E) to confirm that co-localization could be observed with thio-S (Fig. 3F) and using a marker for lysosomes (Lamp-1) (Fig. 3G). Intracellular α-synuclein deposits indeed co-localized with thio-S and Lamp-1 (Figs. 3H,I, arrows), which was not observed in WT tissue (Fig. 3J). Similar colocalization was observed with autophagosomes (LC3-I positive) (Supplementary Fig. 1E).

Extraction of lysosomes/autophagosomes from TWI and WT brains followed by staining with thio-S, anti-α-synuclein antibody, and anti-LAMP-1 or anti-LC3-I antibodies revealed the co-localization of thio-S+ protein aggregates and α-synuclein in lysosomes and autophagosomes preparations from TWI (Fig. 3K,L, white arrows), but not in organelle preparations from WT brains (Supplementary Fig. 1F). Specificity of these findings was confirmed using secondary controls (Supplementary Fig. 1C,D). Tandem mass spectrometry revealed a high concentration of psychosine in the lysosomal/autophagosomal fraction as compared to the total brain amount in TWI, while organelle fractions from WT brains had only negligible amounts of psychosine (Fig. 3M). In immunoblots of fractionated vesicles (Fig. 3N, uncropped blots in Supplementary Fig. 18E–H), high-molecular-weight aggregates of α-synuclein were more readily visible and prevalent in the TWI lysosomal/autophagosomal fractions than those from WT mice, consistent with our previous work that found increased accumulations of insoluble forms of high-molecular-weight α-synuclein in Krabbe’s brains^1^. The higher concentration of LAMP-1 and LC3-I in the fractions from TWI mice than in those from WT mice suggests that GALC deficiency leads to an increase in brain lysosomes/autophagosomes, consistent with results from the immunofluorescence analysis (Fig. 3A–C). Finally, ultrastructural analysis to visualize the aggregated material within autophagosomes/lysosomes identified fibrous material within the limiting membrane of apparent autophagic vacuoles (Fig. 3O, within arrows), and was confirmed to contain α-synuclein via immunogold staining (anti-α-synuclein + anti-IgG-Au antibodies) (Fig. 3P,Q). Secondary controls with TWI tissue showed no non-specific binding (Fig. 3R). Taken together, these results support the notion that *in vivo* interaction between psychosine and α-synuclein can occur due to subcellular segregation of the two molecules within the autophagic/lysosomal pathway.

### Psychosine interacts with α-synuclein *in vitro* and *in vivo*

**Figure 4:**
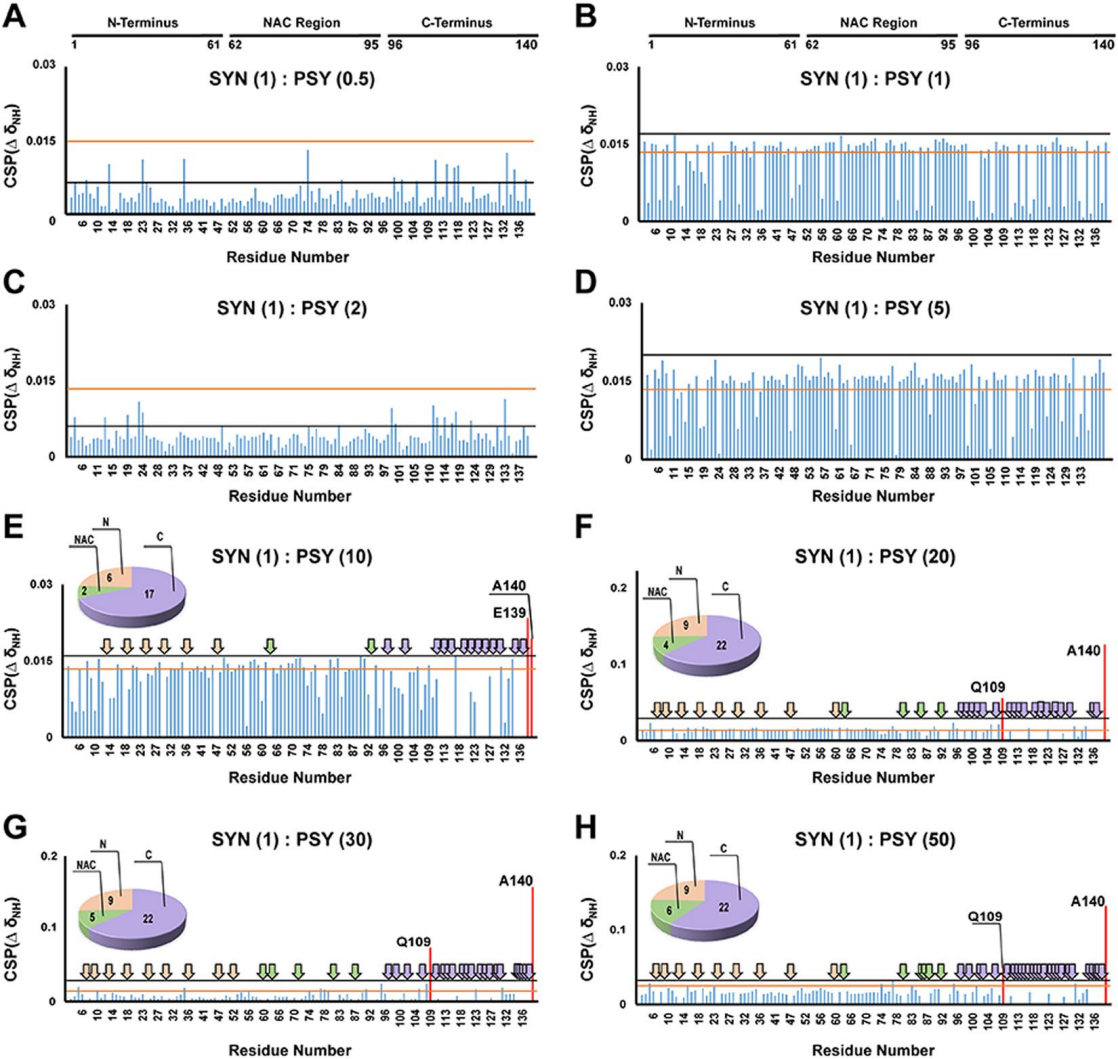
Psychosine interaction with α-synuclein at different concentrations. CSPs in full-length α-synuclein (20 µM) with psychosine indicated no significant effects at the following ratios: α-synuclein (1) psychosine (0.5) (**A**); α-synuclein (1):psychosine (1) (**B**); α-synuclein (1) psychosine (2) (**C**); α-synuclein (1) psychosine (5) (**D**). Significant CSPs and line-broadening of signals were observed at the following molar ratios: α-synuclein (1) psychosine (10) (**E**); α-synuclein (1) psychosine (20) (**F**); α-synuclein (1) psychosine (30) (**G**); α-synuclein (1) psychosine (50) (**H**). The black horizontal line represents the CSP mean plus one SD. The orange horizontal line represents the maximum CSP due to spectral variability between different α-synuclein samples. CSPs higher than both the black and orange lines were considered significant and marked in red. The broadened-beyond-detection signals were assigned by arrows marked in pink in the N-terminus, green in the NAC region, and purple in the C-terminus. The number of signals that were broadened are presented in a pie chart. PSY = psychosine; SYN = α-synuclein.

To address the possibility that psychosine directly interacts with α-synuclein, we employed nuclear magnetic resonance (NMR) spectroscopy. A titration experiment with 20 µM recombinant ^15^N-enriched α-synuclein and a range of concentrations of psychosine was initially performed at neutral pH (7.4) in PBS. At α-synuclein:psychosine (syn:psy) molar ratios of 1:0.5, 1:1, 1:2, and 1:5, no significant chemical shift perturbations (CSPs) were observed in ^15^N HSQC experiments (Fig. 4A–D). However, significant signal attenuation, primarily in the C-terminus, was observed at syn:psy ratios 1:1, 1:2, and 1:5. (Supplementary Fig. 2A–D,I). This change in intensity can result from binding of psychosine, a change in the conformation of α-synuclein, protein aggregation, or a combination of these processes. However, intensity changes arising from protein aggregation are unlikely, since neither significant signal-broadening nor CSPs were observed at these ratios. Thus, at syn:psy ratios of 1:1, 1:2, and 1:5, psychosine appears to have a very weak binding effect, with possible dynamic changes to α-synuclein. Increasing psychosine to a syn:psy molar ratio of 1:10 induced two CSPs in resonances for E139 and A140 and signal-broadening beyond detection predominantly in the negatively charged C-terminus but also in the N-terminal and NAC regions (Fig. 4E). Further increases in psychosine concentration (syn:psy ratios of 1:20, 1:30 and 1:50) led to additional line-broadening and CSPs in amide resonances of residues Q109 and A140 (Fig. 4F–H). Concurrently, signal intensity analysis at ratios 1:10, 1:20, 1:30, and 1:50 showed the greatest changes in the C-terminus, with less significant changes in the NAC region and the N-terminus of α-synuclein (Supplementary Figs 2A–D, S2I). Note that the change in mean intensity in the C-terminus occurred at the 1:5 molar ratio, while the mean intensity began to change in the NAC region and the N-terminus at 1:10 and 1:20 ratios, respectively, indicating that the initial binding event is at the C-terminus, followed by changes in the NAC region and N-terminus.

**Figure 5:**
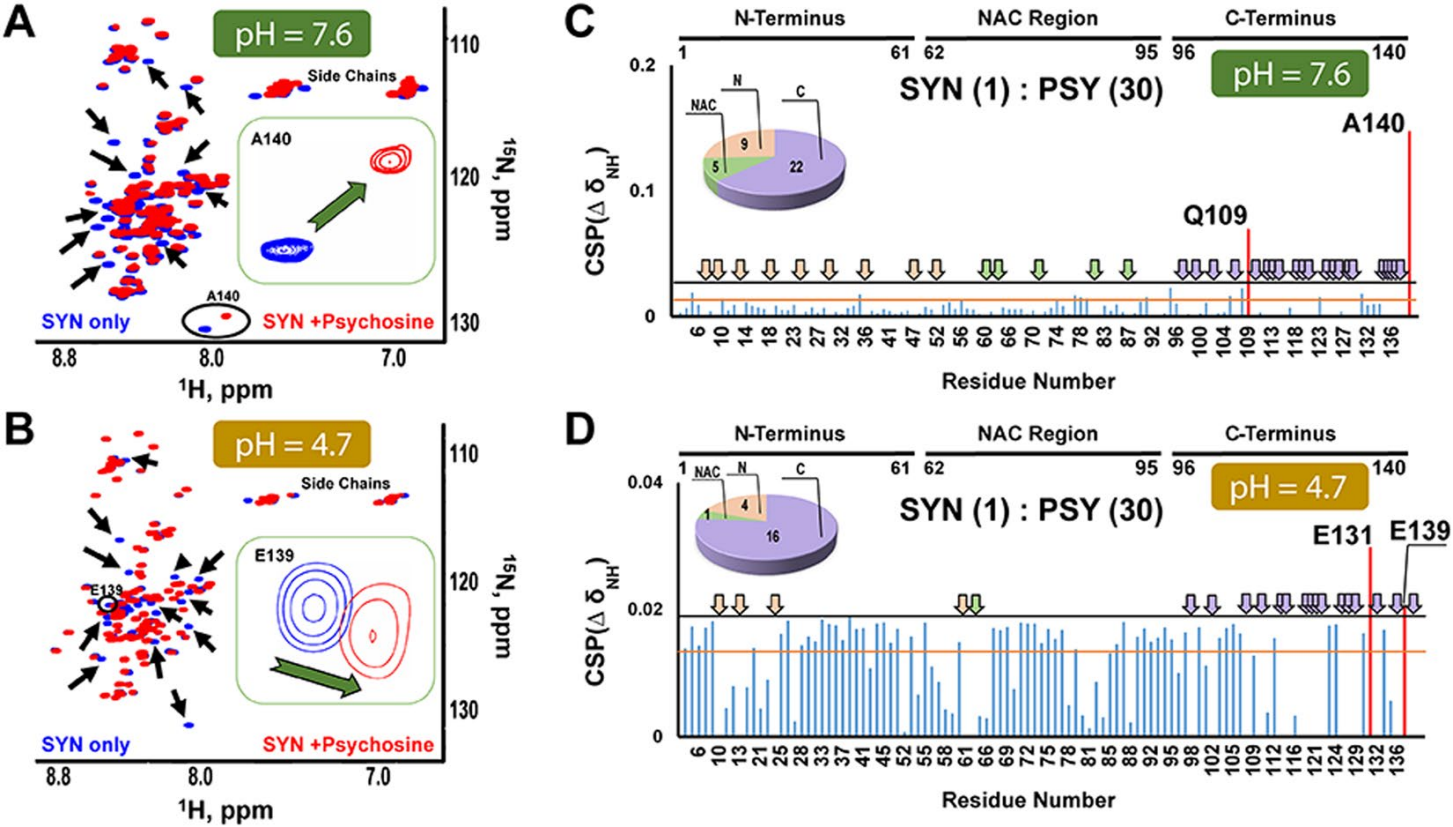
Psychosine interacts with α-synuclein at cytoplasmic (neutral) and lysosomal (acidic) pH. Overlays of ^1^H ^15^N-HSQC NMR spectra of α-synuclein (blue) with α-synuclein (1) psychosine (30) (red) at pH 7.6 (**A**) and α-synuclein (1) psychosine (30) (red) at pH 4.7 (**B**). Examples of chemical shift changes are circled and highlighted in a box. Black arrows indicate areas of signal broadening. The CSPs were measured from the spectra α-synuclein (1) psychosine (30) (red) at pH 7.6 (**C**), and α-synuclein (1) psychosine (30) (red) at pH 4.7 (**D**). The black horizontal line represents the mean CSP plus one SD. The orange horizontal line represents the maximum CSP due to spectral variability between different samples of α-synuclein. CSPs higher than both the black and orange lines were considered significant and are marked in red. The broadened- beyond-detection signals were assigned by arrows marked in pink in the N-terminus, green in the NAC region, and purple in the C-terminus. The number of signals that were broadened are presented in a pie chart. PSY = psychosine; SYN = α-synuclein.

In KD, psychosine is found not only in lipid rafts^33^, but also in lysosomes and in the extra-lysosomal environment (cytoplasm or/and extracellular matrix)^54,55^. These environments differ in their pH (lysosomal pH is ~4.7 and cytoplasmic pH is ~7.6)^55^. Based on the *in vivo* co-localization results (Fig. 3A–R), we tested whether α-synuclein and psychosine interact at acidic (lysosomes) as well as neutral (autophagosomes) pH by repeating the NMR experiments for the syn:psy molar ratio of 1:30 (Fig. 5A,B) at acidic (lysosomal) pH of 4.7. Psychosine interacted with α-synuclein, producing a similar pattern of signal broadening with significant CSPs in resonances for the C-terminal residues E131 and E139 (Fig. 5C,D). Additionally, the change in the mean intensity was similar in the acidic and neutral pH (Supplementary Fig. 3A,B). Thus, within the range of tested pH, cationic psychosine interacts preferentially with the anionic C-terminus, causing signal line-broadening, CSPs, and changes in signal intensities in this region. The line-broadening can result from either exchange between bound and free α-synuclein on the intermediate time-scale, or from reduced backbone dynamics in α-synuclein due to binding of psychosine, which may be further exacerbated by aggregation of psychosine or α-synuclein. Because the signals for α-synuclein do not reappear when it is saturated with psychosine, the intermediate exchange between psychosine-bound and free α-synuclein is unlikely, and the interaction with psychosine is of low affinity. Analysis of the cooperativity of psychosine binding by calculating the Hill coefficient from changes in chemical shifts of Q109 and A140 (residues with the highest CSPs) in the titration of α-synuclein (Supplementary Fig. 4A,B) revealed an average Hill coefficient of 5 based on two fits (Supplementary Fig. 4C,D), indicative of cooperative binding of 5 monomers of psychosine to α-synuclein^56^. Thus, we propose that there are two or more binding events for psychosine. The lowest limit of Kd for the formation of a complex between psychosine and α-synuclein determined by the analysis of signal intensities during the titration is 292 ± 95 µM (Supplementary Fig. 2J). Because several psychosine molecules bind α-synuclein and the interaction is cooperative, the true Kd is difficult to determine. Positive cooperative binding of psychosine might alter exchange between α-synuclein conformations, resulting in the observed line-broadening effects and leading to protein aggregation. Accelerated aggregation of recombinant α-synuclein in the presence of psychosine was previously demonstrated in our thio-S fibrillization assay^1^.

Next, we evaluated the interaction between psychosine and α-synuclein *in vivo* and whether it correlates with *in vitro* results, using human α-synuclein immunoprecipitated (IP) from whole brain lysates from three KD patients and one healthy infantile control patient. Homogenization and washes were performed in detergent-free conditions to avoid disruption of interactions between α-synuclein and the bound lipids. IP was performed using a specific antibody against human α-synuclein and using control IgG antibodies. Background levels of psychosine non-specifically bound to the IgG fraction were subtracted from psychosine measurements in the α-synuclein pulldown to calculate the amount of psychosine specifically bound to α-synuclein (Supplementary Table 1). All three human KD patients’ IPs revealed a detectable level of psychosine above the non-specific binding observed in the IgG fraction. While tissue from the healthy infantile control contained detectable levels of psychosine, no psychosine was detected in pulled-down α-synuclein above control levels. Immunoblotting to confirm the specific pulldown of α-synuclein (Supplementary Fig. 5, uncropped blots in Supplementary Fig. 18I) revealed α-synuclein in the α-synuclein antibody fraction but not when mouse IgG was used.

To further support the feasibility of psychosine-α-synuclein interaction *in vivo*, we measured the syn:psy molar ratio within total cell lysates and also within lysosome/autophagosome extracts from TWI and WT mice by measuring psychosine levels in total cellular lysate and lysosome/autophagosome fractions followed by immunoblotting an equal volume of lysate and lysosome/autophagosome extract for α-synuclein content. α-Synuclein was quantified by comparing the levels in each fraction to a standard curve of known α-synuclein protein run concurrently and subjected to quantitative immunoblotting (Supplementary Fig. 6, uncropped blots in Supplementary Fig. 18J,K). In the total cell lysate, the syn:psy ratio was 1:0.34 (n = 3, SD = 0.079) for TWI and 1:0.005 (n = 3, SD 0.0014) for WT mice, whereas syn:psy ratios of 1:4.73 (n = 3, SD = 1.24) and 1:0.018 (n = 3, SD = 0.0069) were detected in the TWI and WT lysosomal/autophagic fractions, respectively. Although the TWI lysosome/autophagosome extracts ratio is below the NMR syn:psy ratio of 1:10 found to show an effect, we previously showed that lower ratios of syn:psy can still lead to fibrillation for a longer time (96 hours)^1^. Moreover, signal intensity analysis showed that the effect of psychosine on the C-terminus is already observable at a molar ratio of 1:5 (Supplementary Fig. 2I). Our measurements represent the global ratio across all brain regions, which are known to have regional differences in the distribution of both psychosine and α-synuclein^1^. For example, the cortex has a much lower level of psychosine and protein misfolding, while the midbrain and brainstem correlate with high psychosine and higher incidence of protein misfolding^1^. Thus, although whole brain tissue was used to ensure sufficient starting material to extract lysosomes/autophagosomes, local ratios of syn:psy are likely to be higher or lower, probably explaining the regional distribution of protein misfolding.

### Psychosine hydrophilic clusters binding the C-terminus of α-synuclein can expose the NAC region and induce aggregation-prone conformations

Psychosine, an amphiphilic molecule, is known to form toxic aggregates^55^. Using ligand-based NMR approaches (WaterLOGSY), we found that psychosine can form hydrophilic clusters in the absence of α-synuclein and that the hydrophilic epitope of psychosine is involved in the binding upon addition of α-synuclein (Suppl. Results SR1, Supplementary Fig. 7). Comparison of the mechanism of psychosine interaction with α-synuclein to known binding modes of other amine-based compounds such as spermine and spermidine, which promote α-synuclein aggregation by binding the C-terminus and exposing the NAC region, showed that these polyamines caused significant spectral changes that were shared with psychosine but with distinct differences (Suppl. Results SR2, Supplementary Figs 7B, 7C, 8 and 9). Additionally, we validated the involvement of the free amine and galactosyl sugar moieties of psychosine in interacting with α-synuclein by comparing the binding modes of several analogues of psychosine and sphingosine (Supplementary Figs 7, 8D). Finally, we found that psychosine clusters can alter the conformational exchange in α-synuclein by changing the relaxation rates of its NMR signals, as further validated by comparing the effects of sphingosine aggregates in the presence/absence of psychosine and by increasing the psychosine concentration above the critical micelle concentration (Supplementary Figs 10–13).

**Figure 6:**
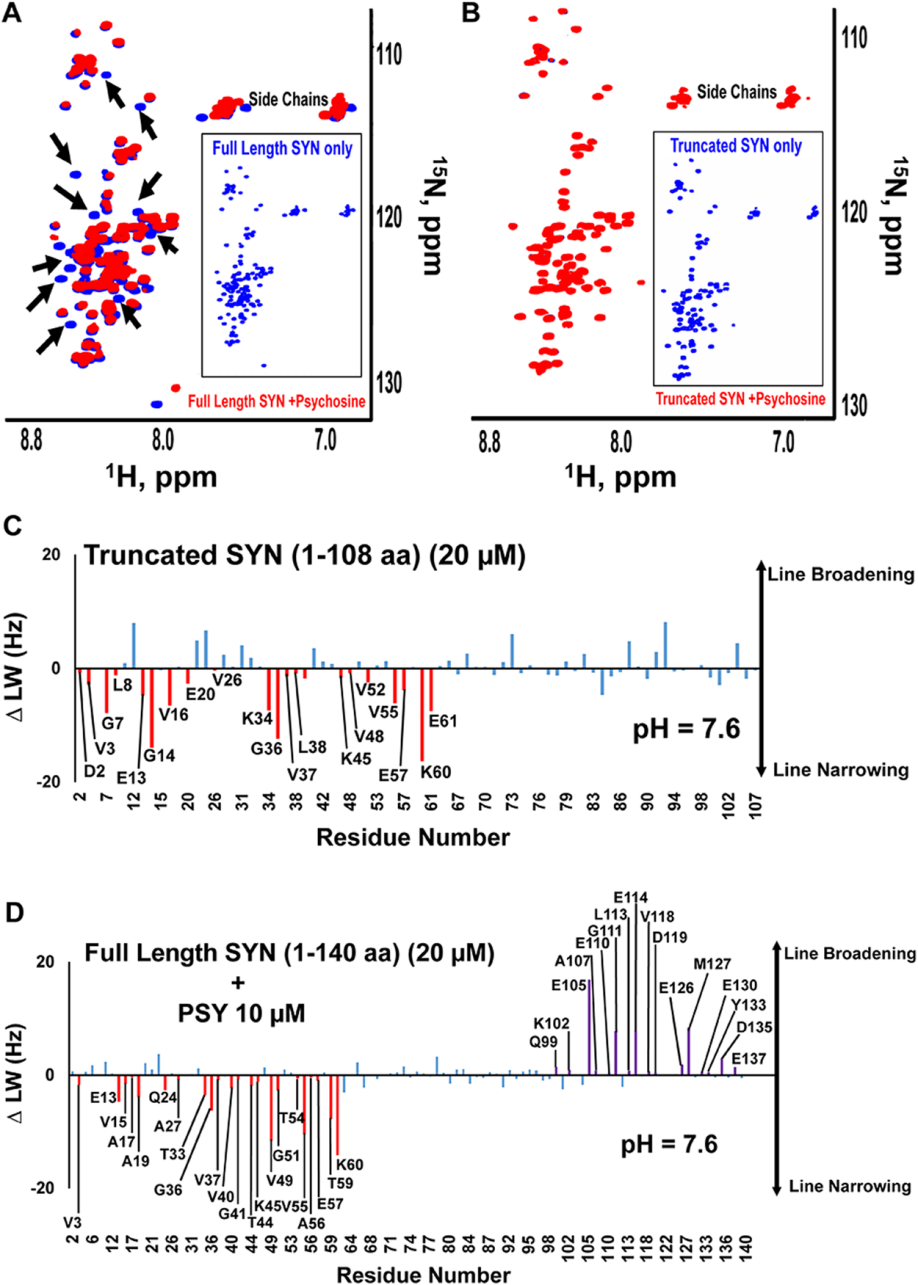
Psychosine interacts with α-synuclein at the C-terminus and truncation of or binding to the C-terminus of α-synuclein releases the N-terminus of α-synuclein. Overlays of ^1^H ^15^N-HSQC NMR spectra of full-length α-synuclein (20 µM, blue) with psychosine (600 µM, red) (**A**) and C-terminus truncated α-synuclein (20 µM, blue) with psychosine (600 µM, red) (**B**). Analysis of ^15^N line-width differences (ΔLW, Hz) between full-length α-synuclein and truncated α-synuclein (**C**) or full-length α-synuclein and α-synuclein in the presence of psychosine (10 µM) (**D**) shows similar line-narrowing in the N-terminus and line-broadening in the C-terminus. Black arrows mark broadening in the signals of full-length α-synuclein in presence of psychosine. Full-length and truncated α-synuclein spectra are shown in boxes separately. Line-narrowing in the N-terminus is marked in red and line-broadening in the C-terminus in purple. SYN = α-synuclein.

Psychosine can cause minimal line-broadening in the N-terminus and the NAC region of α-synuclein (Fig. 4E–H), indicating either direct binding or an indirect effect due to binding the C-terminus. Although psychosine caused CSPs and line-broadening in most residues of the C-terminus of α-synuclein at both lysosomal and cytoplasmic pH, psychosine binding effects were more pronounced in the region between 109–140 aa of α-synuclein (Figs. 5C,D). Thus, we repeated the HSQC experiment at the same concentration of psychosine (600 µM) and using a truncated version of α-synuclein (20 µM) missing the last 32 aa of the C-terminus. Several versions of truncated α-synuclein have been reported in the region between 100 and 140 aa^57,58^. While the truncated version was expected to be more prone to aggregation, under conditions of the NMR experiments and in the timeframe of 15 hours, there was no loss of signals except for the truncated 32 aa of the C-terminus, and incubation for several days was needed to observe significant line-broadening indicative of aggregation (data not shown). The addition of psychosine to truncated α-synuclein (1–108 aa) resulted in no broadening of the signals (Fig. 6A) as compared to the full-length protein (Fig. 6B). This indicates that the C-terminus of α-synuclein is not only the major binding site for psychosine and its hydrophilic clusters, but also a requirement in relaying dynamic changes in the NAC region and the N-terminus leading to aggregation.

α-Synuclein is a disordered protein that can adopt distinct conformations under different conditions, such as pH and lipid composition^7–12,59–64^. For instance, micelle-bound α-synuclein adopts an α-helical structure^65,66^. To address the presence of secondary structure under our experimental conditions, we conducted ^15^N HSQC-NOESY experiments with α-synuclein (20 µM) in the presence and absence of psychosine (600 µM). There were no NOEs characteristic of secondary structure elements (data non-shown). Thus, in our experiments, α-synuclein is likely unfolded and does not have helical segments that are commonly observed in α-synuclein complexes with phospholipid micelles^65,66^.

Several studies have demonstrated long-range interactions between the C-terminus and the N-terminus/NAC region of natively unfolded monomeric α-synuclein^12,59,67^. These interactions allow α-synuclein to exist as an ensemble of compact conformations rather than a randomly unfolded protein. The release of such interactions induces fully extended, open conformations that are susceptible to aggregation^11,12,68^. The equilibrium between the two conformational ensembles can be modulated by polyamines^11,12^. To confirm long-range interactions under our experimental conditions, we explored the effects of truncation of the C-terminus in α-synuclein by analyzing changes in line-widths. The truncation resulted in a marked decrease in the line-widths of signals in the N-terminus, while the NAC region was significantly less affected (Fig. 6C). This line-narrowing suggests that the C-terminus of full-length α-synuclein restricts the conformational dynamics in the N-terminus. Addition of 10 µM psychosine to 20 µM α-synuclein mimicked the line-narrowing effect of the C-terminal truncation, while line-broadening was observed in the C-terminus (Fig. 6D). We suggest that the line-broadening effect at the C-terminus is due to binding of psychosine, and the line-narrowing at the N-terminus occurs due to disruption of the long-range interactions with the C-terminal portion of the protein. This effect was present at all tested concentrations of psychosine in the titration of α-synuclein (Supplementary Fig. 14). Moreover, high psychosine concentrations induced severe line-broadening, causing the signals in the C-terminal and NAC regions to disappear, possibly due to psychosine clustering at the C-terminus and promoting aggregation of α-synuclein.

### Dopamine and carbidopa reverse the pro-aggregation effects of psychosine

**Figure 7:**
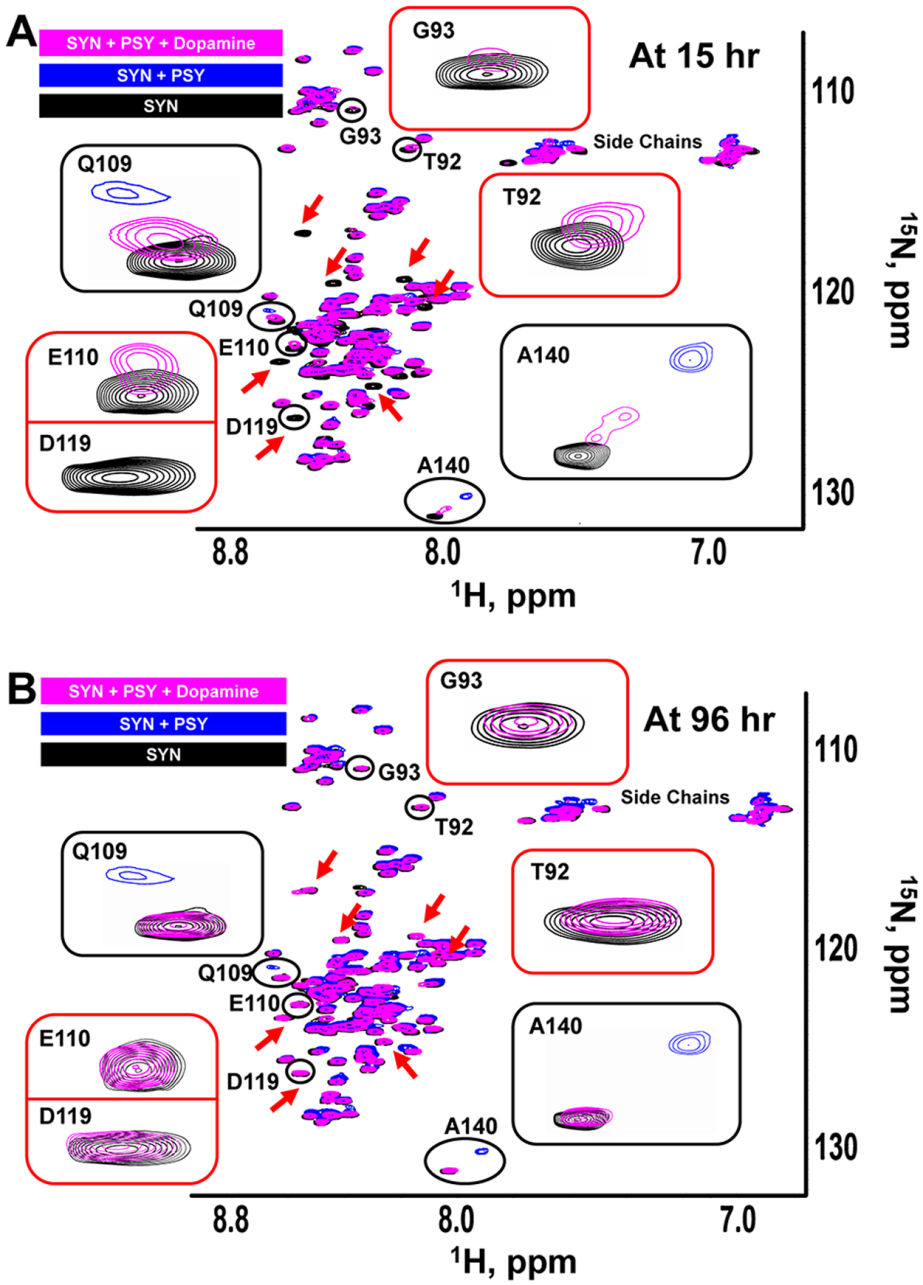
Dopamine blocks psychosine interaction with α-synuclein. Overlays of ^1^H ^15^N-HSQC NMR spectra of full-length α-synuclein (20 µM, black) with psychosine (600 µM, blue) and with psychosine and dopamine (600 µM, magenta) at 15 hours (**A**) and 96 hours (**B**). Black boxes highlight chemical shift changes/reversal. Red boxes highlight signal loss/gain changes. Red arrows show the time-dependent effect of dopamine on signal broadening in the presence/absence of psychosine. PSY = psychosine; SYN = α-synuclein.

Several amine-containing compounds bind α-synuclein and induce either open or closed conformations^69,70^. Dopamine, an amine-containing compound used in the treatment of PD, has been shown to influence α-synuclein aggregation^71–76^. We found that 600 µM dopamine partially reversed the line-broadening and CSPs in 20 µM α-synuclein caused by 600 µM psychosine after a 15-hour incubation, an effect highlighted by chemical shift changes for residues Q109 and A140 as they return to positions seen in control α-synuclein (Fig. 7A). The effect was also evident in the partial gain in intensities for the broadened (lost) signals, such as T92, G93, and E110, although the chemical shifts did not appear at their original position similar to the α-synuclein control sample and some signal was still lost, such as D119 (Fig. 7A). To test whether dopamine can elicit full recovery of the NMR signal similar to the ^15^N labeled HSQC of α-synuclein control sample, we monitored dopamine effects for a prolonged incubation time and under the same conditions (Fig. 7B). At 96 hours, dopamine completely reversed the binding effect of psychosine by abolishing signal broadening for all signals, such as T92, G93, E110, and D119, and the chemical shifts returned to their original positions for all signals, similar to the α-synuclein control sample. Since dopamine and its oxidation products might modulate α-synuclein aggregation^77,78^, we evaluated the chemical stability of dopamine under our conditions by NMR. 1D ^1^H NMR spectra of dopamine did not show any change in chemical shifts or peak intensities up to 105 hours (Supplementary Fig. 15). However, a color change and a precipitate formed on day 16. 1D ^1^H NMR spectra for the soluble fraction showed the presence of dopamine with lowered intensity peaks relative to the NMR spectra recorded at 0.5 hour (Supplementary Fig. 16A,B). 1D ^1^H NMR spectra of the precipitant dissolved in DMSO-d_6_ showed signals corresponding to dopamine oxidation products (Supplementary Fig. 16C). These results confirmed that dopamine was chemically stable under our experimental conditions with incubation times of 15 and 96 hours.

At both incubation times, the effect of dopamine was most pronounced in the C-terminus, the binding site of psychosine hydrophilic clusters. Herrera *et al*. also suggested that dopamine inhibits α-synuclein fibrillization via binding to the protein C-terminus^73^. Additionally, we investigated the role of carbidopa, a drug bearing a hydrazine moiety and used as part of a regimen with levodopa to treat PD. Carbidopa minimizes the peripheral metabolism of levodopa, extending its half-life, reducing its toxicity on dopamine, and enhancing its bioavailability in the brain. Although carbidopa cannot permeate the blood-brain barrier (BBB), we sought to use it as evidence for the therapeutic application of hydrazine-based inhibitors and other amine compounds. At 15 hours of incubation, carbidopa partially reduced the line-broadening of α-synuclein signals in the presence of psychosine, such as K80, G93, V95, and E110, and reversed the chemical shift changes as observed in residue Q109 (Supplementary Fig. 17).

**Figure 8:**
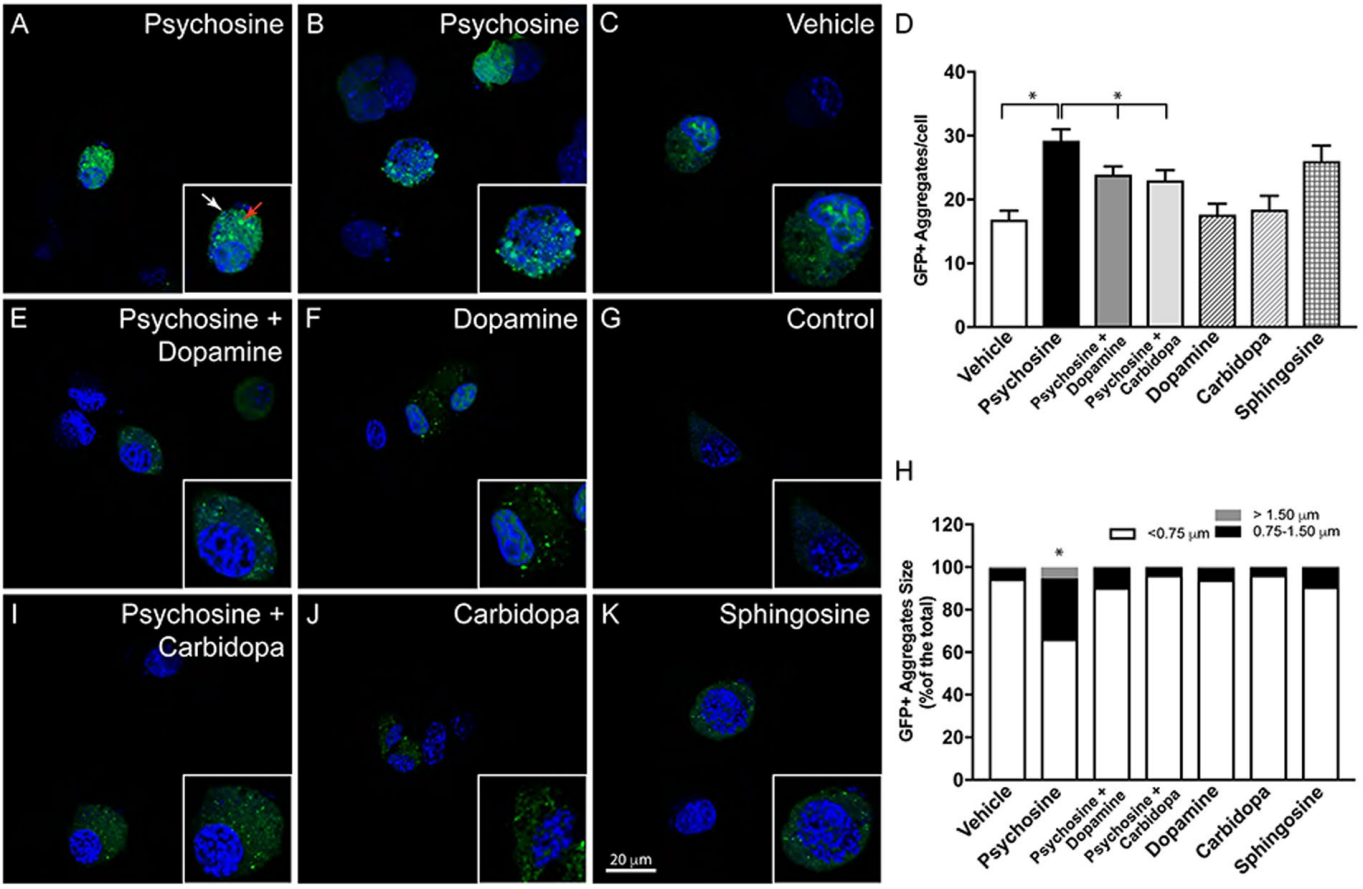
Dopamine and carbidopa prevent psychosine-induced aggregation of α-synuclein. NSC34 motor-neuron cells expressing GN-linkaSYn and a-Syn-GC plasmids^46^ were used to study α-synuclein by bimolecular fluorescence complementation (BiFC). Psychosine (1 μM) was added to NSC34 cell cultures and the formation of fluorescent aggregates of GN-linkaSYn and a-Syn-GC (**A**,**B**) over basal levels in vehicle-treated cells (**C**) was visualized using confocal microscopy. Psychosine facilitated the formation of medium (0.75–1.50 μm, white arrow in A) and large (>1.5 μm, red arrow in **A**) fluorescent aggregates. Addition of 10 μM dopamine to psychosine-medium visually prevented aggregation of BiFC fluorescent α-synuclein (**E**). Bar, 20 μm. Dopamine alone showed insignificant changes in aggregation (**F**). Similar to dopamine, addition of carbidopa in the psychosine-medium blocked the aggregation mediated by psychosine (**I**), with insignificant changes in control conditions (**J**). Sphingosine (1 μM) was used as a control lipid with non-significant differences from basal levels of aggregation. Panel G shows background fluorescence in non-transfected NSC34 cells. Confocal imaging permitted assessment of GFP+ aggregates per cell (**D**) and the distribution of sizes (**H**). Significance, *p < 0.05, ANOVA; mean ± standard error of the mean from 3 independent experiments done in triplicate.

Finally, we examined the capacity of dopamine and carbidopa to influence the formation of α-synuclein aggregates using a bimolecular fluorescence complementation (BiFC) assay to directly image α-synuclein oligomerization and aggregation^46^. Plasmids encoding α-synuclein-venus fusion proteins (kindly donated by Dr. McLean, Mayo Clinic, Jacksonville, FL) were expressed in NSC34 motor-neuronal cells in the presence or absence of psychosine (Fig. 8A–C). Incubation with psychosine greatly favored the formation of fluorescent (Figs. 8A,B, red arrows) large (>0.75 mm in diameter, Fig. 8H) aggregates of α-synuclein, which were significantly more abundant than in vehicle (Fig. 8D) and non-transfected conditions (Fig. 8G). Co-treatment with either dopamine (Fig. 8E,F) or carbidopa (Fig. 8I,J) decreased (Fig. 8D) the formation of large α-synuclein aggregates (Fig. 8H). Together with the NMR analyses, these results suggest that dopamine and carbidopa or their analogs are potential candidates for modulating α-synuclein fibrillization in KD.

## Discussion

Here, we provide the first evidence that a gene therapy approach correcting the deficiency of GALC activity in lysosomes prevents the formation of high-molecular-weight aggregates of α-synuclein, and describe a detailed biophysical study of the molecular interaction between α-synuclein and the GSL psychosine. Furthermore, we present evidence that dopamine and carbidopa are two small compounds that successfully interfere with the capacity of the GSL psychosine to induce fibrillization and aggregation of α-synuclein.

Accumulations of aggregated α-synuclein are believed to contribute significantly to α-synucleinopathies such as PD^79^. Here, we have attempted to isolate the effects of such accumulations on KD via a global knock-out of the *SNCA* gene responsible for α-synuclein, thereby eliminating this potentially harmful proteinopathy. Although assessment of survival, clinical phenotype, body weight, grip strength, and nesting ability revealed significant improvements in TWI that had either one of two alleles or both for SNCA knocked out, these improvements were mild and temporary, as mice displayed severe clinical deficits by their end of life. This is explained by the underlying metabolic deficiency in GALC, which was not corrected in the double TWI/SNCA^−/−^ mice, allowing for the accumulation of psychosine to exert its toxic effects directly via alternative cellular mechanisms^1,35,38–42,51,80^. Thus, direct prevention of α-synuclein pathology in the context of KD is unlikely to yield significant therapeutic benefits without correcting the deficient psychosine metabolism as well.

It is possible that the effectiveness of SNCA removal in KD is limited due to the loss of α-synuclein’s normal cellular function. Depletion of *SNCA* has been shown to cause impairments in the dopaminergic system^45^, and α-synuclein has been suggested to have neuroprotective properties^81^. In our study, TWI with only one copy of *SNCA* removed (TWI/SCNA^+/−^) had a greater increase in survival and a greater number of days in which the clinical scoring was significantly reduced compared to TWI. This suggests that removing one allele may reduce the burden of aggregated α-synuclein due to reduced concentration of the protein, but allow monomeric species to still perform normal cellular functions. Other functions such as grip strength and nesting ability were better corrected in TWI/SNCA^−/−^, suggesting that certain physiological functions are less dependent on the normal function of α-synuclein and benefit more from the complete removal of α-synuclein.

Mutations in glucocerebrosidase, which are responsible for the lysosomal storage disorder Gaucher’s disease, are associated with an increased risk of PD and other synucleinopathies^18,82^. Reducing the accumulation of glucocerebrosides (glucocerebrosidase’s main substrate) has been shown to reduce α-synuclein aberrations, potentially revealing a therapeutic strategy for alleviating some forms of synucleinopathy^83^. Similarly, psychosine has been shown to induce α-synuclein fibrillization *in vitro*^1^ and directly bind to the protein to induce aggregation (this study). Correction of GALC deficiency after gene therapy with AAV9-GALC led to the elimination of α-synuclein high-molecular-weight protein aggregates and thio-S+ amyloid inclusions in the brain throughout the animals’ lifespans. This amelioration of α-synuclein pathology after the correction of GALC deficiency and psychosine accumulation strongly supports the importance of the interaction of psychosine and α-synuclein *in vivo*. These results suggest that correction of GALC activity and the subsequent reduction in psychosine accumulation provide a new therapeutic strategy to intervene in some forms of α-synuclein aggregation.

The observation that cationic psychosine and the negatively charged C-terminus of α-synuclein interact directly sheds light on the potential mechanism of the formation of thio-S reactive aggregates in the brain. Although the initial binding is of low affinity, its positive cooperative nature allows the formation of a stable complex. The amino group and the sugar moiety of psychosine are involved in binding to α-synuclein and in hydrophilic clustering of the sphingolipid on the protein. The acyl chain of the lipid does not contact α-synuclein and is free to engage in interactions with other proteins and lipids. This suggests that the protein can bind psychosine either in solution or in the context of the membrane bilayer.

Hydrophilic clusters of psychosine likely play an important role in stabilizing the “open” aggregation-prone conformation of α-synuclein by exposing the NAC region. This mechanism of α-synuclein aggregation is commonly used by multiple agents and fits the paradigm that aggregation of α-synuclein can be modulated by membrane components and by small molecules^67,84–88^. While psychosine hydrophilic clusters cause significant spectral changes, induce loss of NMR signals, and promote aggregation of α-synuclein, sphingosine aggregates do not promote similar effects. In fact, sphingosine counteracts the effects of psychosine and prevents self-association of α-synuclein. On the other hand, polyamines that do not aggregate bind the C-terminus^11,67^, induce loss of NMR signal, and promote aggregation by affecting protein dynamics^11,12,67,68,84^. This indicates that psychosine and possibly other GSLs have a unique interaction pattern with α-synuclein and can influence protein dynamics differently, warranting further structural investigations. Importantly, the small compounds dopamine and carbidopa can act on already aggregated α-synuclein and lead to its disassembly. To our knowledge, this is the first example of small molecules reversing the effect of psychosine on α-synuclein aggregation.

NMR studies point to the existence of intrinsically unstructured α-synuclein that constantly assumes multiple conformations. External agents can restrict this conformational flexibility, promoting either “open” or “closed” states. Our experiments with dopamine and carbidopa suggest that these “open” and “closed” conformations are not static and can interconvert when conditions are favorable. We propose that dopamine and carbidopa stabilize a closed/aggregation-resistant conformation of the protein that has reduced affinity for psychosine, consistent with the results of Outeiro *et al*.^46^, who showed that dopamine can induce a conformation whereby the C-terminus and N-terminus of α-synuclein are closer together, preventing its fibrillization. However, the mechanism of action of dopamine and carbidopa requires further analyses to address whether dopamine acts on psychosine-induced α-synuclein aggregates by inhibiting the first binding event of psychosine to the C-terminus, or by inhibiting the clustering of psychosine and the second binding effect, or both. Whether this protective effect is only limited to dopamine or can be extended to other small molecules bearing an amino group is also a question that awaits further investigation.

In summary, we have shown that psychosine can interact with α-synuclein *in vivo* and that it can bind directly to the C-terminus of α-synuclein, where the free amino group and the galactosyl sugar moiety of psychosine interact with α-synuclein. We also discovered that psychosine forms hydrophilic clusters (aggregates) in the presence or absence of α-synuclein. Importantly, we show that correction of the lysosomal deficiency of GALC and normalization of psychosine levels by gene therapy^31^ prevents the aggregation of α-synuclein and of thio-S+ material in the context of KD. Moreover, we have identified two small molecules, dopamine and carbidopa, which counteract the effects of psychosine binding to α-synuclein. These results provide a new direction in the understanding of α-synuclein aggregation and offer the possibility to design small molecules that reduce the toxicity of synucleinopathy in KD and other synucleinopathies such as PD.

## Electronic supplementary material

Supplementary Information
