# Supplementary material for: α-Synuclein interacts directly but reversibly with psychosine: implications for α-synucleinopathies
Source: Sci Rep. 2018 Aug 20;8:12462. doi: 10.1038/s41598-018-30808-9 (PMC6102231; doi:10.1038/s41598-018-30808-9)
Supplement: Supplementary file 1 — Supplementary Information [file 41598_2018_30808_MOESM1_ESM.pdf]

α-Synuclein interacts directly but reversibly with psychosine: implications for α-synucleinopathies

Hazem Abdelkarim[1*]; Michael S. Marshall[2*]; Giuseppe Scesa[2]; Rachael A. Smith[2]; Emily Rue[3]; Jeffrey Marshall[2]; Vince Elackattu[2]; Monika Stoskute[2]; Yazan Issa[2]; Marta Santos[2]; Duc Nguyen[2]; Zane Hauck[3]; Richard Breemen[3]; Soledad Celej[4]; Vadim Gaponenko[1]; and Ernesto R. Bongarzone[2,5].

[1]Department of Biochemistry and Molecular Genetics, College of Medicine, University of Illinois at Chicago, Chicago IL 60607; [2]Department of Anatomy and Cell Biology, College of Medicine, University of Illinois at Chicago, Chicago IL 60612; [3]Department of Medicinal Chemistry and Pharmacognosy, College of Pharmacy, University of Illinois at Chicago, Chicago IL 60612. [4]Departamento de Química Biológica Ranwel Caputto, and Centro de Investigaciones en Química Biológica de Córdoba, CIQUIBIC, CONICET, Universidad Nacional de Córdoba, Córdoba, Argentina. [5]Departamento de Química Biológica, IQUIFIB, Universidad Nacional de Buenos Aires, Buenos Aires, Argentina.

[*] These authors contributed equally to this work.

Authors for correspondence:
Dr. Vadim Gaponenko, Department of Biochemistry and Molecular Genetics, University of Illinois at Chicago, 900 S. Ashland Ave., Chicago, IL 60607, USA. e-mail: vadimg@uic.edu.

Dr. Ernesto R. Bongarzone, Department of Anatomy and Cell Biology, College of Medicine, University of Illinois at Chicago. 808 S. Wood St M/C 512, Chicago. IL. USA. email: ebongarz@uic.edu

Supplementary Results (SR)

SR1. Psychosine forms hydrophilic clusters in the presence or absence of α-synuclein

Psychosine, an amphiphilic molecule (**Supplementary Fig. 6A**), aggregates at acidic and neutral pH, and these aggregates mediate its toxic effect [1]. We tested the ability of psychosine to aggregate under the conditions of NMR experiments using the WaterLOGSY NMR approach [2] to determine whether psychosine forms hydrophilic aggregates in the absence of α-synuclein. In WaterLOGSY, water polarization is transferred via the protein-ligand or ligand-ligand complex, such that portions of small molecules involved in binding a large protein show a positive signal in NMR, while non-binding portions show a negative signal. In the one-dimensional reference spectrum, the signal for the amino group was detected only at pH 4.7 due to slow exchange with the solvent under these conditions (**Supplementary Fig. 6B**). At pH 7.6, resonance for the galactosyl moiety and amino group overlapped, producing a signal at 3.35 ppm (**Supplementary Fig. 6C**). At pH 4.7 and 7.6, methylene and methyl groups (hydrophobic region) were detected at 2.5 and 1.2 ppm, respectively (**Supplementary Fig. 6B and 6C**). The WaterLOGSY spectra show that the amino and galactosyl moieties of psychosine, but not the methylene and methyl groups, participate in psychosine aggregation at acidic and neutral pH (**Supplementary Fig. 6D and 6E**). These findings suggest that psychosine exists in equilibrium between single molecules and aggregated forms at acidic and neutral pH, and that its self-association employs hydrogen bonds between the amino and hydroxyl groups in the galactosyl structure. Addition of α-synuclein to psychosine at pH 4.7 and 7.4 did not prevent the hydrophilic clustering of psychosine (**Supplementary Fig. 6F and 6G**), but the presence of α-synuclein at lysosomal and cytoplasmic pH increased the intensity of signals corresponding to the hydrophilic region of psychosine (**Supplementary Fig. 6H** and **6I**). The observed increase in the WaterLOGSY transfer efficiency when α-synuclein is present excludes the possibility that psychosine aggregates initiate interaction with α-synuclein, since α-synuclein binding primarily to psychosine aggregates would have shifted the equilibrium between monomeric and oligomeric psychosine to the monomers, and the WaterLOGSY signal would have decreased. This suggests that single molecules of psychosine can use their amino and galactosyl groups for self-association and for interaction with α-synuclein. Although psychosine does not engage its acyl chain in binding α-synuclein, it is possible that the acyl chain plays a stabilizing role in psychosine aggregates.

SR2. The free amino group and galactosyl sugar moiety of psychosine mediate binding to α-synuclein at the C-terminus

To compare the mechanism of psychosine interaction with α-synuclein to the known binding modes of other amine-containing compounds, we studied spermine and spermidine, which are polyamines that promote α-synuclein aggregation by preventing formation of the closed conformation [3-5]. The amino groups of polyamines bind the negatively charged C-terminus of α-synuclein and hinder its interaction with the positively charged N-terminal portion of the protein, exposing the aggregation-prone NAC region [5]. The polyamines, added at a 30-fold molar excess, caused CSPs and line-broadening in the ^{15}N HSQC spectrum of α-synuclein (**Supplementary Fig. 7B, 7C, 8A, and 8B**). While addition of psychosine resulted in line-broadening mostly in the C-terminus

(**Supplementary Fig. 7A and 8C**), spermine and spermidine caused signal line-broadening in the N-terminus and the NAC region (**Supplementary Fig. 8A and 8B**), with similar effects on signal intensity changes (**Supplementary Fig. 9C and 9D**). Spermine and spermidine caused significant changes in signal intensities in the N-terminus and the NAC region, as evidenced by the higher average intensity values in both regions as compared with the C-terminus (**Supplementary Fig. 9C and 9D**). The difference may be related to the greater number of amino groups in the polyamines than in the single amino group in psychosine, enabling a single polyamine molecule to interact with multiple carboxyl groups in α-synuclein and restricting the conformational dynamics in the protein. Alternatively, the galactosyl moiety in psychosine might contribute to binding α-synuclein, although the addition of a 30-fold molar excess of modified psychosine, in which the amino group was blocked by an acetyl moiety, caused neither CSPs nor signal broadening in the ^{15}N HSQC spectrum of α-synuclein (**Supplementary Fig. 7D**). These results suggest that the amino group in psychosine is essential for binding α-synuclein, while the galactosyl and acyl groups do not directly interact with this protein. Analysis of α-synuclein incubated with a 30-fold molar excess of n-hexanoyl sphingosine or sphingosine, a degradation product of psychosine that has the amino group and the acyl chain but lacks the galactosyl moiety, showed that like n-acetyl-psychosine, n-hexanoyl sphingosine did not cause CSPs or signal line-broadening in the ^{15}N HSQC spectrum of α-synuclein (**Supplementary Fig. 7E**). Sphingosine induced CSPs in the C-terminal residue E126 (**Supplementary Fig. 8D**), but also in the N-terminal and NAC regions of α-synuclein (**Supplementary Fig. 7F and 8D**), confirming the role of the amino group in binding the protein. However, unlike psychosine, sphingosine did not cause severe signal line-broadening.

Because both psychosine and sphingosine bind α-synuclein but differ in their ability to cause NMR signal line-broadening, we tested whether the unique spectral characteristics of the psychosine-α-synuclein complex might reflect protein binding to large lipid micelles. Both psychosine and sphingosine form micelles but differ in their critical micellar concentrations (CMC). For psychosine, the CMC is 1.26 mM at pH 4.0 [1] and for sphingosine, only in the range of 18 μM to 112 μM [6,7]. Sasaki et al. (2009) determined that sphingosine CMC is 0.99 μM at pH 7.2. We find that psychosine aggregates under our experimental conditions at acidic and neutral pH (**Supplementary Fig. 6**). To test whether sphingosine also aggregates, we recorded 1D ^{1}H-NMR spectra at 10 μM and 600 μM, the latter concentration used in experiments with α-synuclein (**Supplementary Fig. 10A**). The spectra of 10 μM and 600 μM sphingosine differed, with chemical shift changes and line-broadening observed at the higher concentration (**Supplementary Fig. 10B and 10C**). Similarly, a control comparison of 1D ^{1}H spectra of 5, 10, and 600 μM psychosine identified the presence of aggregates even at 10 μM (**Supplementary Fig. 11**). These results suggest that sphingosine and psychosine aggregate at 600 μM. Sphingosine binds α-synuclein but, unlike psychosine, does not cause severe line-broadening in NMR spectra. To test whether the latter rests in a lower binding affinity of sphingosine for α-synuclein than for psychosine, we performed a competition experiment, in which sphingosine and psychosine were added at equimolar concentrations of 600 μM to 20 μM of α-synuclein and ^{15}N HSQC spectra were recorded (**Supplementary Fig. 12A**); the severe line-broadening characteristic of psychosine binding was not observed (**Supplementary Fig. 12B and 12C**). This implies that, although the affinity for sphingosine is low, as evidenced by fast exchange on the NMR

time-scale in the spectra of the sphingosine-α-synuclein complex (**Supplementary Fig. 7F and 8D**), it exceeds the affinity for psychosine. Alternatively, sphingosine might interact with psychosine and alter its ability to bind α-synuclein. To provide further evidence that severe line-broadening in the spectra of the psychosine-α-synuclein is not due to interactions with high-molecular- weight lipid micelles, we performed two ^{15}N HSQC NMR experiments, both at a psychosine-to-α-synuclein molar ratio of 1:30, but at different concentrations for psychosine (1200 and 2100 μM, below and above the CMC, respectively) and α-synuclein (40 and 70 μM) (**Supplementary Fig. 13B and 13C**). The spectra did not differ significantly with respect to the extent of NMR signal line-broadening (**Supplementary Fig. 13**), supporting the conclusion that severe line-broadening in the spectra of psychosine-α-synuclein is not due to lipid micelles. These results led to an alternative hypothesis that psychosine binding alters conformational exchange in α-synuclein, changing the relaxation rates of its NMR signals.

Legends to Supplementary Figures and Table

Supplementary Figure 1. Quality control for immunofluorescence imaging, lysosomal enrichment, α-synuclein antibody specificity and purification. Immunofluorescence secondary controls, incubated with thio-S, displayed no non-specific staining in brain tissue **(A, B)**. Fluorescent secondary controls in isolated lysosomes/autophagosomes displayed no non-specific staining or auto-fluorescent material **(C,D)**. Immunofluorescent markers for α-synuclein (blue) and autophagosome (LC3-**I**, red) were stained in TWI P40 brain tissue, demonstrating co-localization (**E**). Lysosomes isolated from wild-type (WT) mice contained lysosomes that co-localized with α-synuclein (yellow arrows) and lysosomes with no α-synuclein **(**white arrow); however none were observed to have thio-S-positive material **(F)**. Quality control for enrichment of lysosomes demonstrated an increase in lysosomal marker Lamp-1 compared to input in both the Twitter and WT preparations **(G)**. Quality control for α-synuclein antibody (preadsorved with soluble α-synuclein protein and using α-synuclein knockout brain lysates) confirmed specificity of binding to monomers and high molecular weight forms of α-synuclein. **(H)** Quantitative PCR analysis of α-synuclein mRNA. **, $p<0.01$; ****$p<0.0001$. **(I)** Purification of recombinant α-synuclein was analyzed by SDS-PAGE and probed with monoclonal antibodies against human α-synuclein. For improved resolution, the purified protein was electrophoresed on 16% Tricine gel and stained with Coomassie blue **(J)**. α-Syn = α-synuclein. Scale bars: 10 μm for **(E)**. Uncropped blots displayed in supplementary fig. 16.

Supplementary Figure 2. Analysis of α-synuclein signal intensities in the presence of psychosine. Changes in intensities of signals of α-synuclein (20 μM) in the presence of 10 μM psychosine **(A),** 20 μM psychosine **(B)**, 40 μM psychosine **(C)**, 100 μM psychosine **(D)**, 200 μM psychosine **(E)**, 400 μM psychosine **(F)**, 600 μM psychosine **(G)**, and 10 μM psychosine were examined (**H**). Mean differences in signal intensity in each region of α-synuclein are shown (**I**). Dissociation constants of psychosine binding to full-length α-synuclein (mean ± SEM) using the C-terminus region mean intensity differences at the above concentrations of psychosine **(J)**. The black horizontal line represents the mean intensity (+SD) in the presence of psychosine. The orange horizontal line represents the mean intensity (+SD) between different samples of α-synuclein. Intensities higher than both the black and orange lines were considered significant. Mean intensities calculated for each region of α-synuclein are shown as a green, red, and purple line for the N-terminal, NAC, and C-terminal regions, respectively. The mean intensity analysis shows that the α-synuclein C-terminus region is the most affected area by psychosine. Loss of signal is assigned as 100% change; SYN =α-synuclein; PSY = psychosine; N = N-terminus of α-synuclein, and C = C-terminus of α-synuclein.

Supplementary Figure 3. Analysis of α-synuclein signal intensities in the presence of psychosine at acidic and neutral pH and in the presence of polyamines. Changes in α-synuclein (20 μM) signal intensity in the presence of 600 μM psychosine at pH 4.7 **(A)**, 600 μM psychosine at pH 7.6 **(B),** 600 μM spermine **(C),** and 600 μM spermidine **(D)** are shown. The black and orange horizontal lines represent the mean intensity (+ 1 SD) in the presence of psychosine and between different samples of α-synuclein, respectively. Intensities higher than both the black and orange lines were considered significant. Mean

intensities were calculated for each region of α-synuclein and shown as green, red and purple lines for the N-terminal, NAC and C-terminal regions, respectively.

Supplementary Figure 4. Assessing cooperativity of psychosine binding to α-synuclein ^{15}N-HSQC chemical shift changes for residues Q109 and A140 in α-synuclein titrated with psychosine were assessed. Overlaid spectra for α-synuclein in the presence of increasing psychosine concentrations (0-600 µM) are colored as follows: black (0 µM), dim gray (10 µM), green (20 µM), magenta (40 µM), yellow (100 µM), violet (200 µM), orange (400 µM), and blue (600 µM) for residue Q109 **(A)** and A140 **(B)**. Hill coefficients (mean +/-SD) were calculated based on data for Q109 **(C)** and A140 **(D)** by nonlinear regression fitting of the difference in ^{15}N chemical shifts, using GraphPad Prism 5 - one site-specific binding with Hill slope. Black arrows indicate the directionality of the changes in both ^{1}H and ^{15}N dimensions.

Supplementary Figure 5. Immunoprecipitation quality control. Brain lysate (Input) and immunoprecipitated proteins were immunoblotted for α-synuclein. A control analysis using normal mouse IgG showed no associated α-synuclein. In both the infantile Krabbe's disease and control patient tissue, α-synuclein was detected at levels higher than those in the Input. Uncropped blots displayed in supplementary fig. 16.

Supplementary Figure 6. Immunoblot of α-synuclein standard curve and in vivo levels. Serial dilutions of α-synuclein protein were immunoblotted, along with lysate from Twitter (TW) and wild-type (WT) whole-brain lysates and enriched lysosomes/autophagosomes. A best-fit curve was applied to the known concentrations of α-synuclein and used to estimate the level of α-synuclein within whole-cell lysate and lysosomal/autophagic fractions. Uncropped blots displayed in supplementary fig. 16.

Supplementary Figure 7. Psychosine aggregates in the presence or absence of α-synuclein at cytoplasmic (neutral) and lysosomal (acidic) pH. Chemical structure of psychosine **(A)**. 1D NMR spectra of 600 µM psychosine (blue) at pH 4.7 **(B)** and pH 7.6 **(C);** WaterLOGSY spectra of 600 µM psychosine (red) at pH 4.7 **(D)** and pH 7.6 (**E**); and WaterLOGSY spectra of 600 µM psychosine and 20 µM α-synuclein (green) at pH 4.7 **(F)** and pH 7.6 are shown **(G)**. Signal Intensity analysis of the hydrophilic signal of psychosine (asterisk) in the absence (red) or presence (green) of α-synuclein at pH 4.7 (**H**) and pH 7.6 (**I**). The physicochemical properties and signal assignments of psychosine are indicated on the spectra.

Supplementary Figure 8. The sugar moiety and free amino group pf psychosine are essential for the interaction with α-synuclein. Overlays of ^{1}H ^{15}N-HSQC NMR spectra of α-synuclein (20 µM, blue) with psychosine (600 µM, red) **(A)**, spermine (600 µM, red) **(B)**, spermidine (600 µM, red) **(C)**, N-acetyl-psychosine (600 µM, red) **(D)**, N-hexanoyl-D--sphingosine (600 µM, red) **(E)**, and D-sphingosine (600 µM, red) **(F)**. Residues exhibiting statistically significant CSPs are marked. Examples of chemical shift changes are circled and highlighted in a box. Black arrows indicate areas of signal broadening. The chemical structures of sphingolipids and polyamines are shown in their respective ^{1}H ^{15}N-HSQC NMR spectra. SYN = α-synuclein.

Supplementary Figure 9. Psychosine has a characteristic binding pattern in comparison with polyamines and sphingosine. CSPs of residues in full-length α-

synuclein (20 μM) with spermidine (600 μM) **(A),** spermidine (600 μM) **(B)**, psychosine (600 μM) **(C)**, and D-sphingosine (600 μM) **(D)**. The black horizontal line represents the mean CSP (+ 1 SD). The orange horizontal line represents the maximum CSP due to variability in spectra of α-synuclein. CSPs higher than both the black and orange lines were considered significant and are marked in red. Broadened-beyond-detection signals were assigned an arbitrary negative CSP value and marked in red. The broadened-beyond-detection signals were assigned by arrows and are marked in pink, green, and purple in the N-terminus, the NAC region, and the C-terminus, respectively. The number of broadened signals are presented in a pie chart. SYN = α-synuclein.

Supplementary Figure 10. Sphingosine forms aggregates at 600 μM. 1D ^{1}H NMR spectra of sphingosine at 600 μM (red) and 10 μM (blue); boxes highlight differences **(A).** Long black arrows highlight spectral differences in the range of 0.8 to 1.8 ppm between sphingosine at 600 μM (red) and 10 μM (blue) **(B),** while short black arrows highlight spectral differences in the range of 3.3 to 3.7 ppm between sphingosine at 600 μM (red) and 10 μM (blue) **(C)**. D-SPN = D-sphingosine.

Supplementary Figure 11. Assessment of psychosine aggregation at neutral pH (7.6). 1D ^{1}H NMR spectra of psychosine were recorded at 2400 μM (green) **(A)**, 600 μM (red) **(B)**, 200 μM (purple) **(C)**, 10 μM (blue) **(D)**, and 5 μM (black) **(E)**. Signal assignments of psychosine are marked on the spectra. Black arrows indicate differences in spectra between several concentrations of psychosine. PSY = psychosine.

Supplementary Figure 12. Sphingosine abates psychosine-induced line-broadening and chemical shift changes in α-synuclein NMR signals. Overlays of ^{1}H ^{15}N-HSQC NMR spectra of full-length α-synuclein (20 μM blue) with sphingosine and psychosine (600 μM, red) at pH=7.6 **(A)**. Examples and patterns of chemical shift changes of full-length α-synuclein (20 μM, blue) with sphingosine and psychosine (600 μM, red) at pH=7.6; black arrows show directionality of the chemical shift changes **(B)**. CSPs of residues in full-length α-synuclein (20 μM) with sphingosine and psychosine at 600 μM **(C)**. The black horizontal line represents the CSP mean (+ 1 SD). The orange horizontal line represents the maximum CSP due to spectral variability among different α-synuclein samples. CSPs higher than both the black and orange lines were considered significant and are marked in red. SYN = α-synuclein; D-SPN = D-sphingosine; and PSY = psychosine.

Supplementary Figure 13. Psychosine causes similar CSPs and line-broadening patterns of full-length α-synuclein signals at higher concentrations. CSPs of residues in full-length α-synuclein (20 μM) with psychosine (600 μM) **(A)**; in full-length α-synuclein (40 μM) with psychosine (1200 μM) **(B)**; and in full-length α-synuclein (70 μM) with psychosine (2100 μM) **(C)**. The black horizontal line represents the mean CSP (+ 1 SD). CSPs higher than the black line were considered significant and are marked in red. The broadened-beyond-detection signals are marked by pink, green, and purple arrows in the N-terminus, the NAC region, and in the C-terminus, respectively. The pie chart shows the number of broadened signals. PSY = psychosine; SYN = α-synuclein.

Supplementary Figure 14. Psychosine affects line-widths of full-length α-synuclein. ^{15}N line-width differences (ΔLW, Hz) for α-synuclein (20 μM) in the presence of psychosine at 20 μM **(A),** 40 μM **(B)**, 100 μM **(C)**, 200 μM **(D)**, 400 μM **(E),** 600 μM **(F)**,

and 1000 µM **(G)** show line-broadening in the C-terminus signals (purple) and line-narrowing in the N-terminus (red) of α-synuclein. Signals broadened beyond detection in **D**,**E**,**F** and **G** were excluded from the analysis. SYN = α-synuclein; PSY = psychosine.

Supplementary Figure 15. Dopamine is chemically stable up to 105 hours. 1D ^{1}H NMR spectra of dopamine were recorded at 600 µM in PBS and 10% D_2O, pH 7.6 at **(A)** 0.5 hour (blue) **(B)**, 24 hours (red) **(C)**, 48 hours (green) **(D)**, 72 hours (magenta) **(E)** 96 hours (black), and **(F)** 105 hours (orange). The spectra show the two distinct regions of dopamine: the aromatic region (6.0 – 7.0 ppm) and the methylene groups (2.0 -4.0 ppm). Chemical shifts or peak intensities remained unchanged.

Supplementary Figure 16. Detection of Dopamine oxidation products. 1D ^{1}H NMR spectra of dopamine were recorded at 600 µM in PBS and 10% D_2O, pH 7.6 at **(A)** 0.5 hour (red) **(B)**, 16 days (green). A decrease in intensity was observed due precipitation of solid material. **(C)** 1D ^{1}H NMR spectra of dopamine precipitates were recorded in100% DMSO-d_6 at day 17 (blue). Black arrows indicate signals that correspond to dopamine oxidation products. Solvent peaks were marked in the spectra.

Supplementary Figure 17. Carbidopa blocks psychosine-induced aggregation of α-synuclein. Overlays of ^{1}H ^{15}N-HSQC NMR spectra of full-length α-synuclein (20 µM, blue) with psychosine (600 µM, red), and with psychosine and carbidopa (600 µM, green) at 15 hours. Black boxes/circles highlight chemical shift changes/reversal and signal loss/gain changes. PSY = psychosine; SYN = α-synuclein.

Supplementary Figure 18. Uncropped western blots. Blots **A-D** used for quantification as shown in Fig. 2L, α-synuclein monomer and HMW bands shown. Blot **E** used in Fig. 3N, α-synuclein monomer and HMW bands shown. Blots **F-H** used in Fig. 3N, actin, LC3-I, and Lamp-1 shown respectively. Blot **I** used in Supplementary Fig. 5, α-synuclein shown. Blots **J** and **K** used in Supplementary Fig. 6, α-synuclein shown.

Supplementary Table 1. Immunoprecipitation of α-synuclein-associated psychosine. Brain lysate from post-mortem tissue of two infantile KD patients, one late-onset KD patient, and a control infantile patient was immunoprecipitated using antibodies against α-synuclein or normal mouse IgG as a control. Psychosine levels detected in the control IgG immunoprecipitates were subtracted from those of the α-synuclein immunoprecipitates to exclude measurement of any non-specific binding of the lipid to the IP beads; the resulting amount of psychosine was considered sufficient for interaction with α-synuclein to be extracted from the input lysate.

Supplementary References

1 Orfi, L., Larive, C. K. & LeVine, S. M. Physicochemical characterization of psychosine by 1H nuclear magnetic resonance and electron microscopy. *Lipids* **32**, 1035-1040 (1997).

2 Dalvit, C., Fogliatto, G., Stewart, A., Veronesi, M. & Stockman, B. WaterLOGSY as a method for primary NMR screening: practical aspects and range of applicability. *J Biomol NMR* **21**, 349-359 (2001).

3 Antony, T. *et al.* Cellular polyamines promote the aggregation of alpha-synuclein. *J Biol Chem* **278**, 3235-3240 (2003).

4 Fernandez, C. O. *et al.* NMR of alpha-synuclein-polyamine complexes elucidates the mechanism and kinetics of induced aggregation. *The EMBO journal* **23**, 2039-2046 (2004).

5 Bertoncini, C. W. *et al.* Release of long-range tertiary interactions potentiates aggregation of natively unstructured alpha-synuclein. *Proc Natl Acad Sci U S A* **102**, 1430-1435 (2005).

6 Contreras, F. X., Sot, J., Alonso, A. & Goni, F. M. Sphingosine increases the permeability of model and cell membranes. *Biophys J* **90**, 4085-4092 (2006).

7 Deguchi, H., Yegneswaran, S. & Griffin, J. H. Sphingolipids as bioactive regulators of thrombin generation. *J Biol Chem* **279**, 12036-12042, (2004).

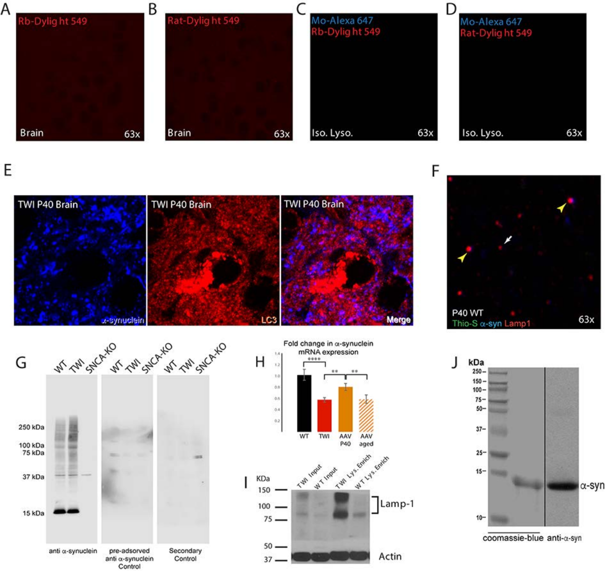
A
Rb-Dylig ht 549
Brain
63x
B
Rat-Dylig ht 549
Brain
63x
C
Mo-Alexa 647
Rb-Dylig ht 549
Iso. Lyso.
63x
D
Mo-Alexa 647
Rat-Dylig ht 549
Iso. Lyso.
63x
E
TWI P40 Brain
α-synuclein
TWI P40 Brain
LC3
TWI P40 Brain
Merge
F
P40 WT
Thio-S α-syn Lamp1
63x
G
WT TWI SNCA-KO
WT TWI SNCA-KO
WT TWI SNCA-KO
250 kDa
100 kDa
75 kDa
37 kDa
15 kDa
anti α-synuclein
pre-adsorved anti α-synuclein Control
Secondary Control
H
Fold change in α-synuclein mRNA expression
1.4
1.2
1.0
0.8
0.6
0.4
0.2
****
**
**
WT
TWI
AAV P40
AAV aged
I
KDa
TWI Input
WT Input
TWI Lys. Enrich
WT Lys. Enrich
150
100
75
50
37
Lamp-1
Actin
J
kDa
250
150
100
75
50
37
25
15
10
α-syn
coomassie-blue
anti-α-syn

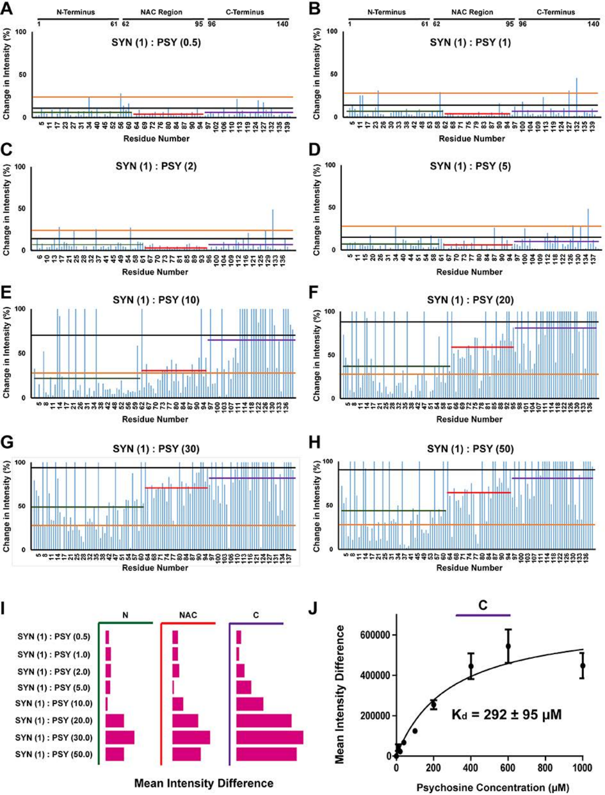

A
B
C
D
E
F
G
H
I
J
N-Terminus
NAC Region
C-Terminus
1
61
62
95
96
140
SYN (1) : PSY (0.5)
SYN (1) : PSY (1)
SYN (1) : PSY (2)
SYN (1) : PSY (5)
SYN (1) : PSY (10)
SYN (1) : PSY (20)
SYN (1) : PSY (30)
SYN (1) : PSY (50)
Change in Intensity (%)
Residue Number
N
NAC
C
SYN (1) : PSY (0.5)
SYN (1) : PSY (1.0)
SYN (1) : PSY (2.0)
SYN (1) : PSY (5.0)
SYN (1) : PSY (10.0)
SYN (1) : PSY (20.0)
SYN (1) : PSY (30.0)
SYN (1) : PSY (50.0)
Mean Intensity Difference
C
Mean Intensity Difference
Kd = 292 ± 95 μM
Psychosine Concentration (μM)

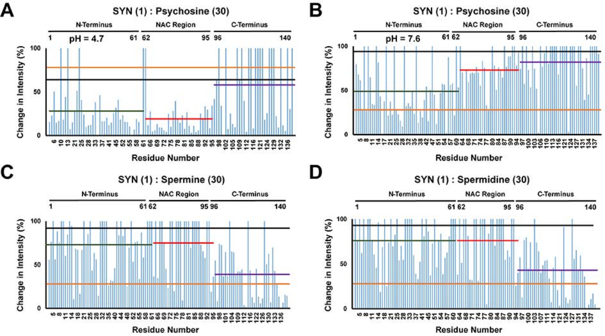

A
SYN (1) : Psychosine (30)
N-Terminus
NAC Region
C-Terminus
1
pH = 4.7
61
62
95
96
140
Change in Intensity (%)
Residue Number
B
SYN (1) : Psychosine (30)
N-Terminus
NAC Region
C-Terminus
1
pH = 7.6
61
62
95
96
140
Change in Intensity (%)
Residue Number
C
SYN (1) : Spermine (30)
N-Terminus
NAC Region
C-Terminus
1
61
62
95
96
140
Change in Intensity (%)
Residue Number
D
SYN (1) : Spermidine (30)
N-Terminus
NAC Region
C-Terminus
1
61
62
95
96
140
Change in Intensity (%)
Residue Number

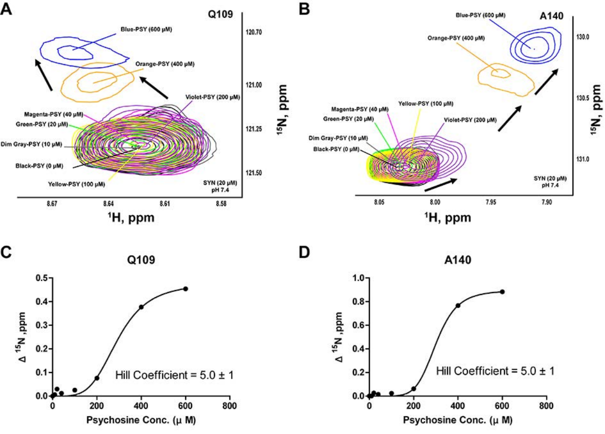
A
Q109
Blue-PSY (600 μM)
Orange-PSY (400 μM)
Violet-PSY (200 μM)
Magenta-PSY (40 μM)
Green-PSY (20 μM)
Dim Gray-PSY (10 μM)
Black-PSY (0 μM)
Yellow-PSY (100 μM)
SYN (20 μM)
pH 7.4
120.70
121.00
121.25
121.50
8.67
8.64
8.61
8.58
1H, ppm
15N, ppm
B
A140
Blue-PSY (600 μM)
Orange-PSY (400 μM)
Magenta-PSY (40 μM)
Yellow-PSY (100 μM)
Green-PSY (20 μM)
Violet-PSY (200 μM)
Dim Gray-PSY (10 μM)
Black-PSY (0 μM)
SYN (20 μM)
pH 7.4
130.0
130.5
131.0
8.05
8.00
7.95
7.90
1H, ppm
15N, ppm
C
Q109
Δ 15N ,ppm
Hill Coefficient = 5.0 ± 1
Psychosine Conc. (μ M)
0.0
0.1
0.2
0.3
0.4
0.5
0
200
400
600
800
D
A140
Δ 15N ,ppm
Hill Coefficient = 5.0 ± 1
Psychosine Conc. (μ M)
0.0
0.2
0.4
0.6
0.8
1.0
0
200
400
600
800

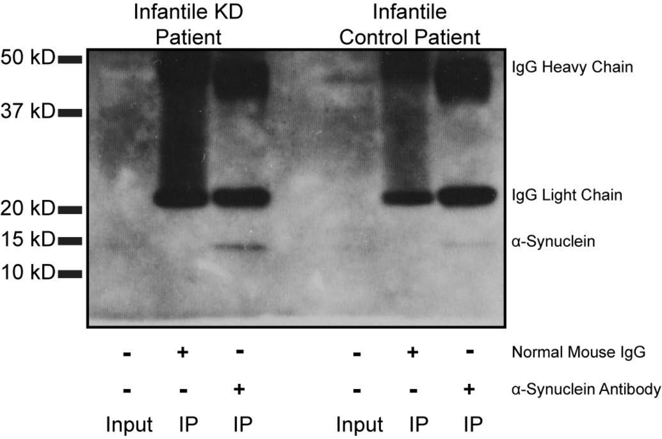
Infantile KD
Patient
Infantile
Control Patient
50 kD
37 kD
20 kD
15 kD
10 kD
IgG Heavy Chain
IgG Light Chain
α-Synuclein
- + - - + -
Normal Mouse IgG
- - + - - +
α-Synuclein Antibody
Input IP IP Input IP IP

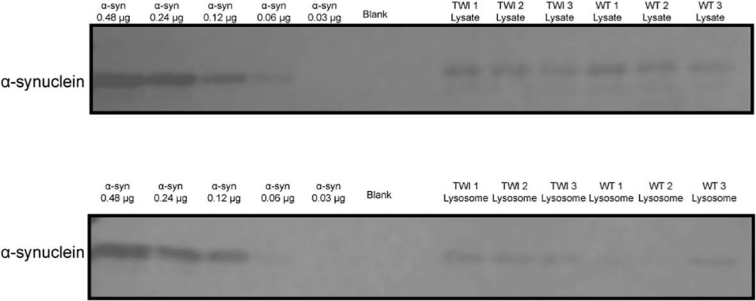
α-syn 0.48 µg
α-syn 0.24 µg
α-syn 0.12 µg
α-syn 0.06 µg
α-syn 0.03 µg
Blank
TWI 1 Lysate
TWI 2 Lysate
TWI 3 Lysate
WT 1 Lysate
WT 2 Lysate
WT 3 Lysate
α-synuclein
α-syn 0.48 µg
α-syn 0.24 µg
α-syn 0.12 µg
α-syn 0.06 µg
α-syn 0.03 µg
Blank
TWI 1 Lysosome
TWI 2 Lysosome
TWI 3 Lysosome
WT 1 Lysosome
WT 2 Lysosome
WT 3 Lysosome
α-synuclein

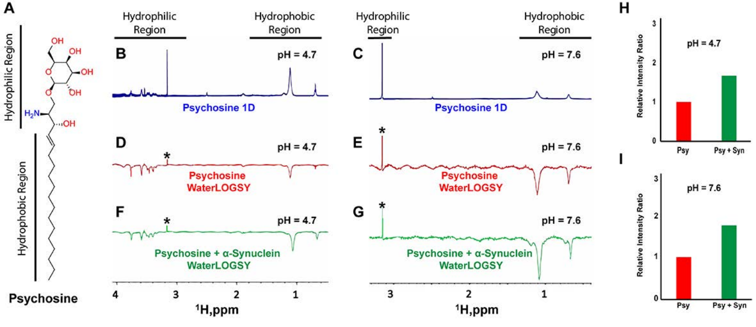
A
Hydrophilic Region
Hydrophobic Region
OH
OH
OH
OH
O
O
H_2N
OH
Psychosine
Hydrophilic Region
Hydrophobic Region
B
pH = 4.7
Psychosine 1D
D
pH = 4.7
*
Psychosine WaterLOGSY
F
pH = 4.7
*
Psychosine + α-Synuclein WaterLOGSY
4
3
2
1
^{1}H,ppm
Hydrophilic Region
Hydrophobic Region
C
pH = 7.6
Psychosine 1D
E
*
pH = 7.6
Psychosine WaterLOGSY
G
*
pH = 7.6
Psychosine + α-Synuclein WaterLOGSY
3
2
1
^{1}H,ppm
H
pH = 4.7
Relative Intensity Ratio
0
1
2
3
Psy
Psy + Syn
I
pH = 7.6
Relative Intensity Ratio
0
1
2
3
Psy
Psy + Syn

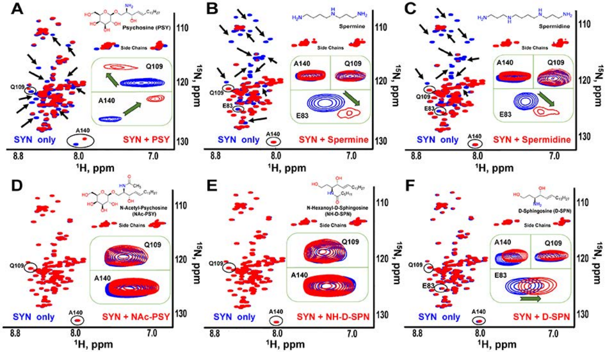
A
Psychosine (PSY)
Side Chains
Q109
A140
SYN only
SYN + PSY
B
Spermine
Side Chains
A140
Q109
E83
SYN only
SYN + Spermine
C
Spermidine
Side Chains
A140
Q109
E83
SYN only
SYN + Spermidine
D
N-Acetyl-Psychosine (NAc-PSY)
Side Chains
Q109
A140
SYN only
SYN + NAc-PSY
E
N-Hexanoyl-D-Sphingosine (NH-D-SPN)
Side Chains
Q109
A140
SYN only
SYN + NH-D-SPN
F
D-Sphingosine (D-SPN)
Side Chains
A140
Q109
E83
SYN only
SYN + D-SPN
¹H, ppm
¹⁵N, ppm
8.8
8.0
7.0
110
120
130

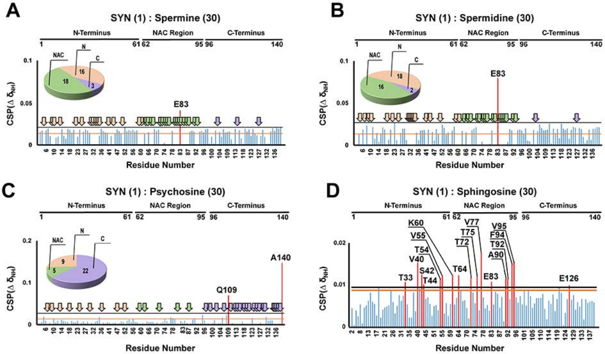

A
SYN (1) : Spermine (30)
N-Terminus
NAC Region
C-Terminus
1
61
62
95
96
140
NAC
N
C
16
18
3
E83
CSP(Δ δNH)
Residue Number
B
SYN (1) : Spermidine (30)
18
16
2
E83
C
SYN (1) : Psychosine (30)
9
5
22
Q109
A140
D
SYN (1) : Sphingosine (30)
K60
V55
T54
V40
S42
T44
T33
T64
V77
T75
T72
E83
V95
F94
T92
A90
E126

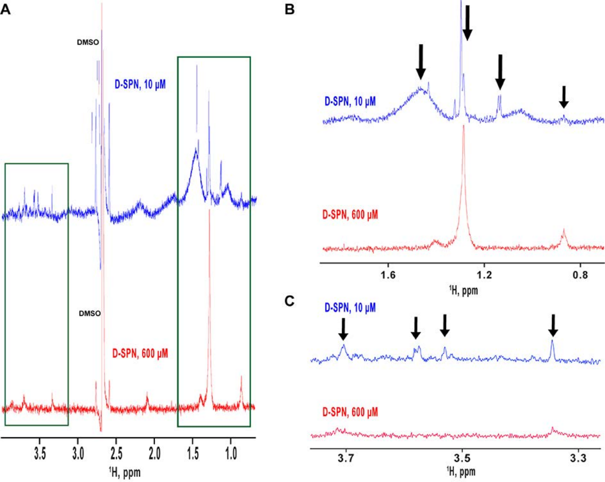
A
DMSO
D-SPN, 10 µM
DMSO
D-SPN, 600 µM
3.5
3.0
2.5
2.0
1.5
1.0
¹H, ppm
B
D-SPN, 10 µM
D-SPN, 600 µM
1.6
1.2
0.8
¹H, ppm
C
D-SPN, 10 µM
D-SPN, 600 µM
3.7
3.5
3.3
¹H, ppm

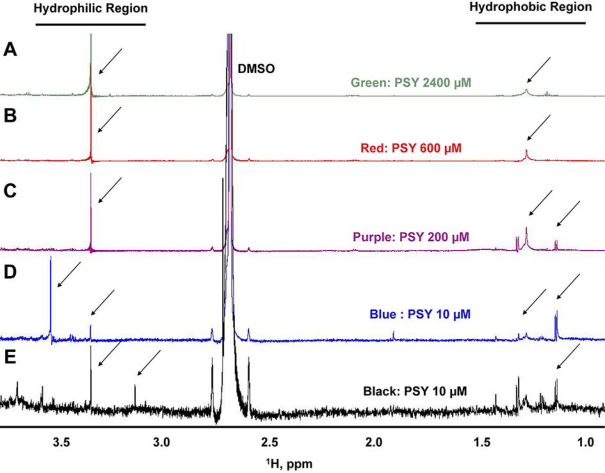
Hydrophilic Region
Hydrophobic Region
A
B
C
D
E
DMSO
Green: PSY 2400 μM
Red: PSY 600 μM
Purple: PSY 200 μM
Blue : PSY 10 μM
Black: PSY 10 μM
3.5
3.0
2.5
2.0
1.5
1.0
1H, ppm

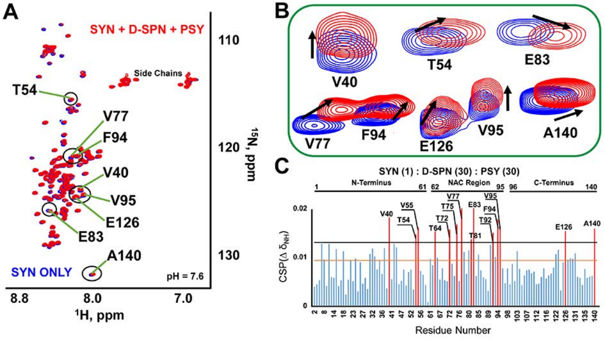

A
SYN + D-SPN + PSY
Side Chains
T54
V77
F94
V40
V95
E126
E83
A140
SYN ONLY
pH = 7.6
110
120
130
15N, ppm
8.8
8.0
7.0
1H, ppm
B
V40
T54
E83
V77
F94
E126
V95
A140
C
SYN (1) : D-SPN (30) : PSY (30)
N-Terminus
NAC Region
C-Terminus
CSP(Δ δNH)
Residue Number

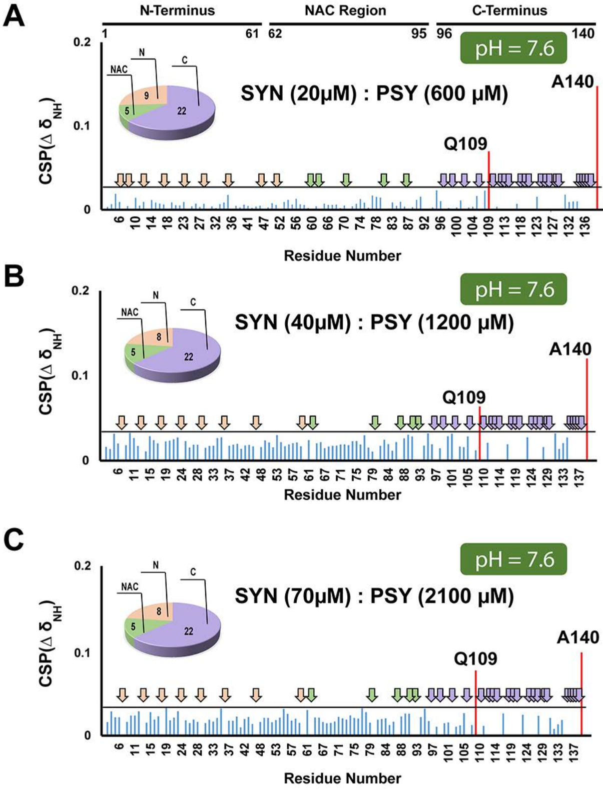
A
N-Terminus
NAC Region
C-Terminus
pH = 7.6
SYN (20μM) : PSY (600 μM)
Q109
A140
CSP(Δ δNH)
Residue Number
B
pH = 7.6
SYN (40μM) : PSY (1200 μM)
Q109
A140
CSP(Δ δNH)
Residue Number
C
pH = 7.6
SYN (70μM) : PSY (2100 μM)
Q109
A140
CSP(Δ δNH)
Residue Number

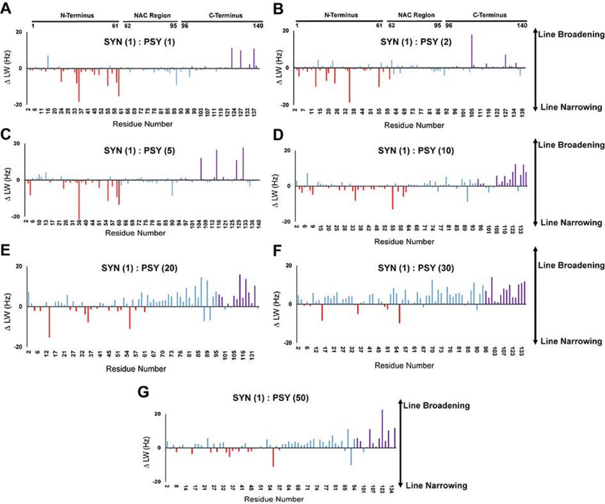
A
N-Terminus
NAC Region
C-Terminus
1
61
62
95
96
140
SYN (1) : PSY (1)
Δ LW (Hz)
Residue Number
B
SYN (1) : PSY (2)
Line Broadening
Line Narrowing
C
SYN (1) : PSY (5)
D
SYN (1) : PSY (10)
E
SYN (1) : PSY (20)
F
SYN (1) : PSY (30)
G
SYN (1) : PSY (50)

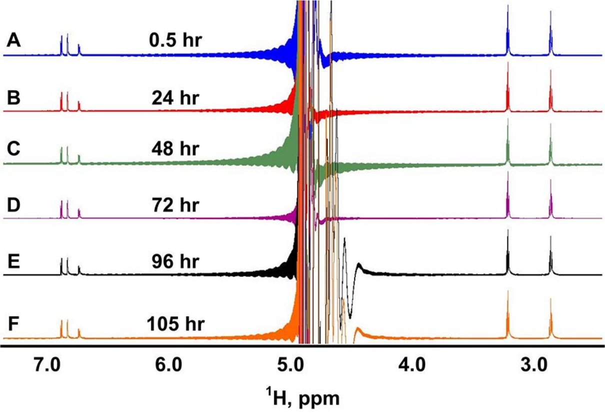
A
0.5 hr
B
24 hr
C
48 hr
D
72 hr
E
96 hr
F
105 hr
7.0
6.0
5.0
4.0
3.0
^{1}H, ppm

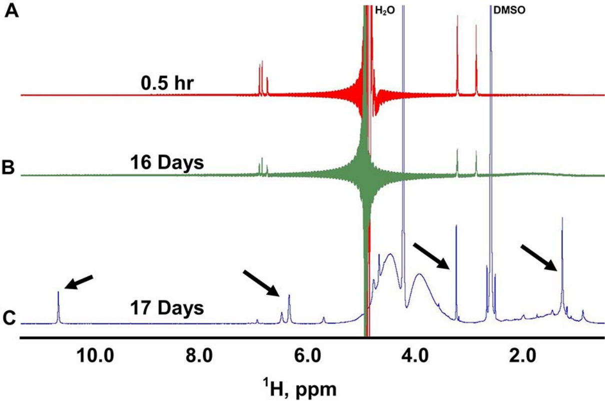

A
B
C
H2O
DMSO
0.5 hr
16 Days
17 Days
10.0
8.0
6.0
4.0
2.0
1H, ppm

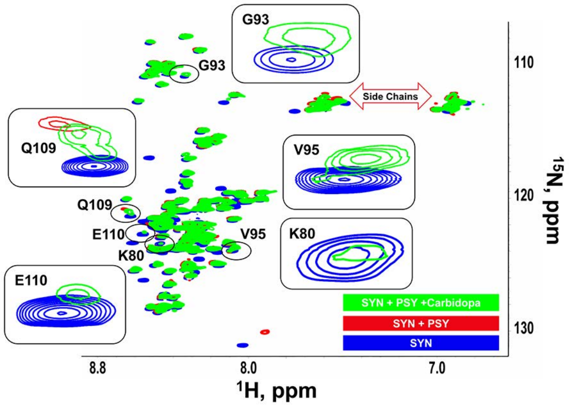
G93
G93
Side Chains
Q109
V95
Q109
E110
K80
V95
K80
E110
SYN + PSY +Carbidopa
SYN + PSY
SYN
110
120
130
^{15}N, ppm
8.8
8.0
7.0
^{1}H, ppm

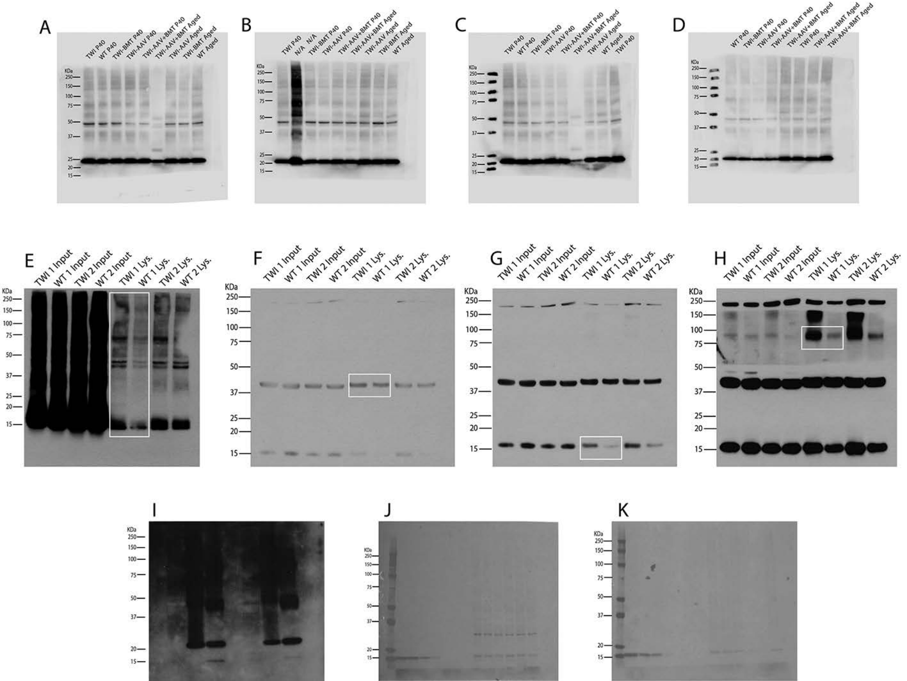
A
TWI P40
WT P40
TWI-BMT P40
TWI-AAV P40
TWI-AAV+BMT P40
TWI-AAV+BMT Aged
TWI-AAV Aged
TWI-BMT Aged
WT Aged
B
TWI P40
N/A
N/A
TWI-BMT P40
TWI-AAV P40
TWI-AAV+BMT P40
TWI-AAV+BMT Aged
TWI-AAV Aged
TWI-BMT Aged
WT Aged
C
TWI P40
WT P40
TWI-BMT P40
TWI-AAV P40
TWI-AAV+BMT P40
TWI-AAV+BMT Aged
TWI-AAV Aged
WT Aged
TWI P40
D
WT P40
TWI-BMT P40
TWI-AAV P40
TWI-AAV+BMT P40
TWI-AAV+BMT Aged
TWI P40
TWI-AAV+BMT Aged
TWI-AAV+BMT Aged
E
TWI 1 Input
WT 1 Input
TWI 2 Input
WT 2 Input
TWI 1 Lys.
WT 1 Lys.
TWI 2 Lys.
WT 2 Lys.
F
TWI 1 Input
WT 1 Input
TWI 2 Input
WT 2 Input
TWI 1 Lys.
WT 1 Lys.
TWI 2 Lys.
WT 2 Lys.
G
TWI 1 Input
WT 1 Input
TWI 2 Input
WT 2 Input
TWI 1 Lys.
WT 1 Lys.
TWI 2 Lys.
WT 2 Lys.
H
TWI 1 Input
WT 1 Input
TWI 2 Input
WT 2 Input
TWI 1 Lys.
WT 1 Lys.
TWI 2 Lys.
WT 2 Lys.
KDa
250
150
100
75
50
37
25
20
15
I
J
K

	Brain Homogenate psychosine (Input) [fmol/mg tissue]	IP psychosine [fmol/mg tissue]	Percentage (%) of Input psychosine associated with IP
Infantile KD patient 1	4710.4	25.70	0.55
Infantile KD patient 2	1755.2	15.43	0.88
Late-onset KD patient	2754.8	0.93	0.03
Infantile control patient	1630.4	Undetectable above background	Undetectable above background
